# Rapid evolution and copy number variation of primate *RHOXF2*, an X-linked homeobox gene involved in male reproduction and possibly brain function

**DOI:** 10.1186/1471-2148-11-298

**Published:** 2011-10-12

**Authors:** Ao-lei Niu, Yin-qiu Wang, Hui Zhang, Cheng-hong Liao, Jin-kai Wang, Rui Zhang, Jun Che, Bing Su

**Affiliations:** 1 State Key Laboratory of Genetic Resources and Evolution, Kunming Institute of Zoology and Kunming Primate Research Center, Chinese Academy of Sciences, Kunming, Yunnan 650223, China; 2 Graduate School of the Chinese Academy of Sciences, Beijing 100039, China; 3 Department of Internal Medicine, Renal Division, Washington University School of Medicine, St. Louis, MO 63110, USA

## Abstract

**Background:**

Homeobox genes are the key regulators during development, and they are in general highly conserved with only a few reported cases of rapid evolution. *RHOXF2 *is an X-linked homeobox gene in primates. It is highly expressed in the testicle and may play an important role in spermatogenesis. As male reproductive system is often the target of natural and/or sexual selection during evolution, in this study, we aim to dissect the pattern of molecular evolution of *RHOXF2 *in primates and its potential functional consequence.

**Results:**

We studied sequences and copy number variation of *RHOXF2 *in humans and 16 nonhuman primate species as well as the expression patterns in human, chimpanzee, white-browed gibbon and rhesus macaque. The gene copy number analysis showed that there had been parallel gene duplications/losses in multiple primate lineages. Our evidence suggests that 11 nonhuman primate species have one *RHOXF2 *copy, and two copies are present in humans and four Old World monkey species, and at least 6 copies in chimpanzees. Further analysis indicated that the gene duplications in primates had likely been mediated by endogenous retrovirus (ERV) sequences flanking the gene regions. In striking contrast to non-human primates, humans appear to have homogenized their two *RHOXF2 *copies by the ERV-mediated non-allelic recombination mechanism. Coding sequence and phylogenetic analysis suggested multi-lineage strong positive selection on *RHOXF2 *during primate evolution, especially during the origins of humans and chimpanzees. All the 8 coding region polymorphic sites in human populations are non-synonymous, implying on-going selection. Gene expression analysis demonstrated that besides the preferential expression in the reproductive system, *RHOXF2 *is also expressed in the brain. The quantitative data suggests expression pattern divergence among primate species.

**Conclusions:**

*RHOXF2 *is a fast-evolving homeobox gene in primates. The rapid evolution and copy number changes of *RHOXF2 *had been driven by Darwinian positive selection acting on the male reproductive system and possibly also on the central nervous system, which sheds light on understanding the role of homeobox genes in adaptive evolution.

## Background

Homeobox genes encode homeobox proteins that play a crucial role in various developmental processes as transcription factors. A key feature of homeobox proteins is the homeodomain, a 60-amino-acid helix-turn-helix DNA-binding domain [1]. Due to their functional importance during development, most of the homeobox genes (especially the homeodomain) are highly conserved at sequence level [1-3]. There have been only a few published examples of rapid evolution of homeobox genes, such as *OdsH *in flies [4], Hox genes in nematodes [5,6], the *Rhox5 *cluster genes in rodents [7-12], and *TGIFLX *and *ESX1 *in primates [13,14]. Here we report a novel case of rapid evolution as well as copy number variation (CNV) of an X-linked reproductive homeobox family gene, member 2 (*RHOXF2*) in primates. This homeobox gene is involved in spermatogenesis and may also play a role in brain function.

**Figure 1:**
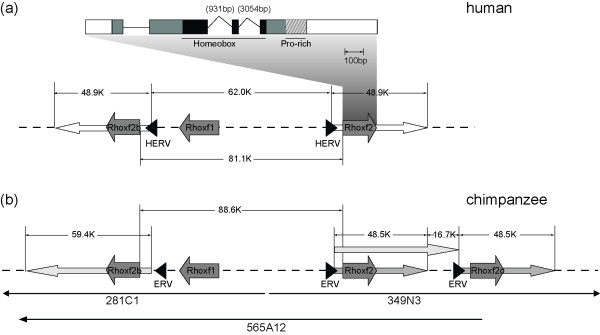
**The schematic genomic structure of *RHOXF2 *gene copies in humans (a) and chimpanzees (b)**. (a) There are two 48.9-Kb segmental tandem repeats containing two copies of the *RHOXF2 *gene, *i.e. RHOXF2 *and *RHOXF2b *in the human genome. (b) Three segmental tandem repeats containing three copies of *RHOXF2 *gene are constructed for chimpanzees according to the three known BAC (bacterial artificial chromosome) clones (281C1, 349N3 and 565A12) of a male chimpanzee (CHORI251). The black triangles indicate endogenous retrovirus (ERV) sequences flanking the breakpoints.

*RHOXF2*, selectively expressed in the testis, was initially identified as a member of the *PEPP *subfamily [15]. The human *RHOXF2 *gene is also named as testis homeobox gene 1 (*THG1*) or human paired-like homeobox protein (*hPEPP2*). It is located on Xq24 and contains 4 exons encoding a 288-amino-acids protein with two functional domains, the homeodomain and the proline-rich domain (figure 1a)[15]. In humans, there are two copies of the *RHOXF2 *gene on Xq24 in a head-to-head orientation, *i.e. RHOXF2 *and *RHOXF2b *[15]. The *Rhox *(reproductive homeobox on the X chromosome) cluster genes in rodents have been considered to be orthologs of human *RHOXF2 *[15]. Recent studies have shown that the human *RHOXF2 *protein is functionally similar to the rodent *Rhox5*, the founding member of the *Rhox *cluster, expressed in the Sertoli cells of the testis and promoting survival and differentiation of the adjacent male germ cells during spermatogenesis [8,16-18]. Similar to the *Rhox *cluster genes in rodents, the human *RHOXF2 *can down-regulate the expression of *Unc5c *and *Pltp*, and up-regulate *Gdap1 *expression in the Sertoli-cell pathway promoting germ cell survival [16,18]. Interestingly, all three down-stream genes directly regulated by *RHOXF2 *in the testis also play important roles in the nervous system. In the brain, *Unc5c *is a receptor of netrin-1, which is important for axonal guidance, neuron migration and proliferation [19,20]. *Pltp *is an important modulator of the signal transduction pathways in the human neurons, and is likely involved in neurodegenerative and inflammatory brain diseases [21,22]. *Gdap1 *is involved in a signal transduction pathway in neuronal development, and is responsible for various Charcot-Marie-Tooth diseases, the most common peripheral neuropathy [23,24].

Due to the function of *RHOXF2 *in spermatogenesis and possibly in the central nervous system, we studied the evolutionary pattern of this homeobox gene in primates, and we observed frequent gene duplications/losses and rapid protein sequence changes. We also performed expression pattern analysis in multiple primate species, and examined between-species and between-paralogs expression divergences. Our evidence suggests that the rapid evolution of *RHOXF2 *at both the sequence and expression levels is likely to have been caused by selection on the male reproductive system and possibly also on the central nervous system.

## Results

### *RHOXF2 *copy number variation in primates

In humans, there are two *RHOXF2 *gene copies on Xq24 in a head-to-head orientation, *RHOXF2 *and *RHOXF2b *(figure 1a) [15]. We sequenced the entire coding sequences (867bp) and about 600bp adjacent noncoding regions (including the entire 185bp intron-1 and the flanking sequences of the exons) in 111 human individuals (including African, European, Melanesian and East Asian; 83 males and 28 females). There are 14 individuals (12 males and 2 females) showing no heterozygous sites in the entire sequenced region. This suggests that these individuals either have two identical or only a single copy of the *RHOXF2 *gene located on the X chromosome.

In the reference human genome, there are two 48.9-Kb segmental tandem repeats containing the two *RHOXF2 *copies (figure 1a). We sequenced the genomic regions covering the breakpoints of the two segmental repeats in 30 human genomic DNA samples (including all individuals without heterozygous site and all individuals subject to qPCR). The data indicated that the breakpoint sequences exist in all individuals (including 14 individuals without heterozygous sites), implying that they all have two copies. This result was further confirmed by genomic DNA real-time quantitative PCR of 9 individuals with heterozygous sites (4 males and 5 females) and 8 individuals without heterozygous sites (7 males and 1 female) (see additional file 1). Using *TKTL1*, an X-linked single copy gene as a control, we found these samples are no difference in *RHOXF2 *genomic DNA quantity (P > 0.05, T test). Collectively, our data demonstrate that the two copies of *RHOXF2 *are fixed in contemporary humans.

To see whether the two-copy structure is conserved in nonhuman primates, we first conducted PCR-based sequencing of the entire coding region of the 16 non-human primate species (table 1). A single copy X-linked gene would have no heterozygous site in males, therefore heterozygous sites in males suggest more than one gene copy. Our results showed that three species (chimpanzees, pig-tailed macaques and rhesus macaques) have heterozygous sites in males, suggesting more than one copy in them. The two leaf monkey species (all females) also have heterozygous sites. Since a single copy X-linked gene would also have heterozygous sites in females due to within-copy polymorphisms, their copy numbers were then determined by genomic DNA qPCR. The other 11 nonhuman primate species do not exhibit any heterozygous sites, implying either a single copy or two/multiple identical copies, which was then tested by genomic DNA qPCR. According to the genome database, there are two copies in the white-tufted-ear marmoset (one intact copy and one incomplete copy due to low quality of the sequence assembly) (http://genome.wustl.edu).

**Table 1:** Summary of the primate species examined for *RHOXF2 *and their sequence divergences from human

Lineage	Common name	**Abbr**.	Scientific name	No. of Individuals	Sex	Protein divergence from human	Ka/Ks	Diverged time from human (my)
Human	Human	HUM	*Homo sapiens*	111/4/7/1*	/	/	/	/

Great Ape	Chimpanzee	CHP	*Pan troglodytes*	6/2/2/1*	/	5.7%	3.44	5
Great Ape	Gorilla	GOR	*Gorilla gorilla*	1	M	5.5%	0.86	7
Great Ape	Orangutan	ORA	*Pongo pygmaeus*	1	M	11.3%	1.14	14

Lesser Ape	Siamang	SIA	*Symphalangus syndactylus*	1	M	17.5%	1.69	18
Lesser Ape	White-browed gibbon	WBG	*Hylobates hoolock*	1/0/0/1*	M	17.9%	1.65	18
Lesser Ape	White-cheeked gibbon	WCG	*Hylobates leucogenys*	1	F	18.3%	1.57	18

Old World Monkey	Grey leaf monkey	GLM	*Trachypithecus phayrei*	1	F	23.5%	1.21	25
Old World Monkey	Black leaf monkey	BLM	*Trachypithecus francoisi*	1	F	22.4%	1.11	25
Old World Monkey	Yunnan golden monkey	YGM	*Rhinopithecus bieti*	1	F	23.0%	1.43	25
Old World Monkey	Douc langur	DL	*Pygathrix nemaeus*	1	M	21.7%	1.22	25
Old World Monkey	Red guenon	RG	*Erythrocebus patas*	1	F	23.9%	1.15	25
Old World Monkey	Stump-tailed macaque	STM	*Macaca arctoides*	1	F	21.1%	0.93	25
Old World Monkey	Pere David's macaque	PDM	*Macaca thibetana*	1	F	21.1%	0.98	25
Old World Monkey	Assam macaque	AM	*Macaca assamensis*	1	M	21.1%	0.98	25
Old World Monkey	Pig-tailed macaque	PTM	*Macaca nemestrina*	1	M	21.1%	0.99	25
Old World Monkey	Rhesus macaque	RM	*Macaca mulatta*	20/2/2/3*	/	21.1%	0.99	25

New World Monkey	White-Tufted-Ear Marmoset	MAR	*Callithrix jacchus*	Database	F	53.4%	1.12	40

* The number of individuals for genomic DNA analysis, brain cDNA expression analysis, testis cDNA expression analysis and multi-tissue expression analysis, respectively.

To identify the *RHOXF2 *gene sequences of individual copies, we cloned and sequenced the PCR products of the five species with heterozygous sites. In pig-tailed macaques and rhesus macaques (all males), we identified two distinct sequences in both species, implying the presence of two copies. In rhesus macaques, there are 6 fixed single nucleotide differences between the two copies and all of them are non-synonymous substitutions located in the proline-rich domain (see additional file 2). We then sequenced 20 rhesus macaque individuals (including both males and females). Interestingly, all 20 rhesus macaques possessed the same two copy sequences without any within-copy polymorphic sites, implying strong functional restriction. The fixed sequence divergence between the two rhesus macaque copies is totally different from the pattern seen in humans, and the two human copies do not exhibit any fixed substitutions.

**Figure 2:**
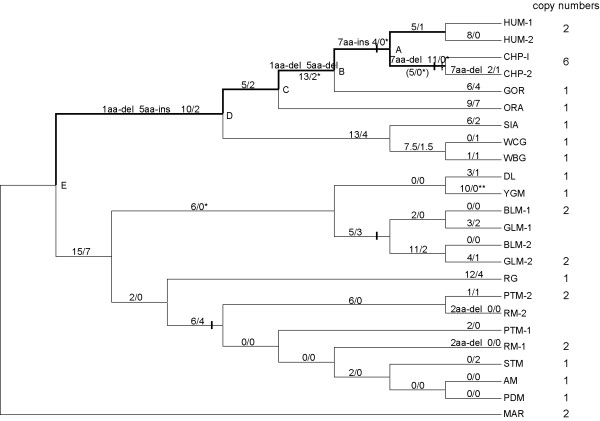
**The phylogenetic tree showing sequence substitution pattern of each primate lineage**. The numbers of non-synonymous and synonymous substitutions (N/S) and the amino-acid insertions-deletions are labeled for each lineage. The labeled "8/0" between HUM-1and HUM-2 only represents the total N/S sites in the human population, and the labeled "7aa-del 2/1" between CHP-1 and CHP-2 just represents the sequence difference between the within-species copies. Using marmoset sequence as outgroup, all internal node sequences were inferred by PAML[36]. The lineages showing Ka/Ks ratios significantly larger than one are denoted by '*' (p < 0.05) or '**' (p < 0.01). The solid bars indicate the duplication events, and the dashed bar on the chimpanzee lineage indicates the uncertainty of number of duplication events. The lineages leading to humans and chimpanzees since the most recent common ancestor of Catarrhines (node E) are shown in thick lines. For the abbreviations of the primate species, refer to table 1.

The grey leaf monkey and the black leaf monkey (all females) also possess two distinct sequences with 16 (14 non-synonymous and 2 synonymous substitutions) and 15 (13 non-synonymous and 2 synonymous substitutions) substitutions respectively. The majority (14/16 for grey leaf monkey, 14/15 for black leaf monkey) of the substitutions are shared between the two species, suggesting that the substitutions are between-copy divergences instead of within-copy polymorphisms. Therefore, grey leaf and black leaf monkeys likely possess two copies of *RHOXF2 *(see additional file 2). Phylogenetic analysis revealed that gene duplication occurred before the two species diverged (figure 2).

**Figure 3:**
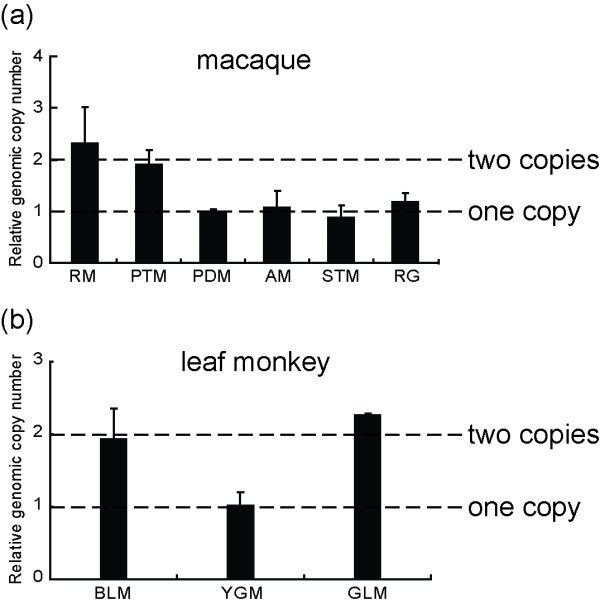
**The result of genomic DNA qPCR in macaques (a) and leaf monkeys (b)**. (a) RM and PTM showed about 2 fold of *RHOXF2 *gene genomic DNA compared to the other macaques. (b) BLM and GLM showed about 2 fold of *RHOXF2 *gene genomic DNA compared to YGM. The single copy X-linked gene, transketolase-like 1 (*TKTL1*) is the reference gene. For the abbreviations of the tested primate species, refer to table 1.

The results of genomic DNA qPCR indicated that the *RHOXF2 *genomic DNA quantities of rhesus macaques and pig-tailed macaques are about twice those of other macaque species with no heterozygous sites (figure 3a). The same result was also seen for leaf monkeys when compared with the Yunnan snub-nosed monkey (no heterozygous sites) (figure 3b). The data further supports that rhesus macaques, pig-tailed macaques, grey leaf and black leaf monkeys possess two *RHOXF2 *gene copies, and there is only one copy in the nonhuman primate species without heterozygous sites.

In chimpanzees, we identified at least 4 different sequences by screening the 26 cDNA clones from the testicle of a male individual, suggesting multiple copies in its genome (see additional file 2 and additional file 3). To explore the detailed genomic structure of *RHOXF2 *gene copies in the chimpanzee, we screened the BAC (bacterial artificial chromosome) library of a male chimpanzee (CHORI251). We obtained 7 positive BAC clones containing the *RHOXF2 *gene sequence, including 281C1, 349N3, 565A12, 602H9, 792F3, 792G12 and 834E8. For three of them (281C1, 349N3 and 565A12), sequences are available in the NCBI database [GenBank: AC145687, AC142344 and AC183597]. Our BAC-end and breakpoint-region re-sequencing confirmed the sequence alignments of the contig (315 kb) containing the three NCBI BAC clones, and 3 copies of *RHOXF2 *gene are located within this contig (figure 1b). Two of the three copies are orthologs of the two human copies according to the synteny of this genomic region. We then partially sequenced the other 4 BAC clones, and we found that 792F3 showed sequence differences from the 3 known BAC clones, an indication of at least one extra copy in chimpanzees. Finally, we performed genomic DNA qPCR and the result showed that the copy number in chimpanzees is about 3 times (3.03 ± 0.65) larger than in humans, suggesting 5-7 copies in chimpanzees. Combining the data from the BAC clones and the cDNA clones from testicle and brain samples (see additional file 2 and additional file 3), there are at least 6 copies in the chimpanzee genome.

To reveal the genomic locations of all these copies in chimpanzees, using BAC clone 281C1 as the probe containing a complete copy of *RHOXF2*, we performed chromosome fluorescence *in situ *hybridization (FISH). The results indicated that all signals are located in the long arm of X chromosome (see additional file 4), suggesting that all the copies are possibly tandem duplications on Xq24, which was partially reflected by the determined genomic structure of the three copies in chimpanzees (figure 1b).

Additionally, we sequenced the entire *RHOXF2 *gene coding region of six chimpanzee individuals (genomic DNA samples). We observed in-del polymorphisms in exon 2 of three chimpanzees, implying that copy number variation might exist in chimpanzees. We also performed cDNA sequencing of testicle samples in chimpanzees, and there are frame-shifting in-del polymorphisms, suggesting that there are non-functional copies (pseudogenes) (see additional file 2).

### Endogenous retrovirus (ERV) sequences and copy number variation

In humans, based on the sequences of male individuals, a total of 12 haplotypes were inferred using PHASE [25]. Interestingly, we did not observe any fixed substitutions between the two human copies. The haplotype pattern indicates that almost all the substitutions are shared by the two copies, suggesting frequent non-allelic homologous recombination between them. Further investigation showed that there are two human endogenous retrovirus (HERV) sequences located at the breakpoint region of the two copies (figure 1). They are ERV3-like sequences, similar to HERV15Yq1 and HERV15Yq2 located at Yq11 in the human genome [26]. It has been shown that the intra-chromosomal homologous recombination between HERV15Yq1 and HERV15Yq2 can mediate duplications and deletions of the azoospermia factor A (AZFa) region on the human Y chromosome, resulting in male infertility [27-29]. We used the HERV15 LTR 787-bp segment sequence as a reference to acquire the HERV sequences located at Xq24 from genome database [30]. It turned out that there is almost no difference (1/783bp) between the two HERV sequences flanking *RHOXF2 *(see additional file 5). Thus, the flanking locations of the two HERV sequences and their high sequence similarity suggest that the HERVs may mediate frequent non-allelic recombinations of the two human copies, similar to the mechanism known for the AZFa region [27,28,30]. In the database of Genomic Variants and 1000-Genomes, low frequency copy number variations (CNVs) were observed in humans covering the *RHOXF2 *gene region (http://projects.tcag.ca and http://browser.1000genomes.org) [31,32], supporting the proposed non-allelic recombinations mediated by the HERVs.

Further analysis indicated that there are also ERV sequences near *RHOXF2 *in the four nonhuman primate species as their whole-genome sequences available for study (chimpanzee, gorilla, orangutan and rhesus macaque) (see additional file 5). Therefore, it is possible that endogenous retrovirus sequences are the key elements causing non-allelic recombinations, resulting in copy number variation among primates. In the marmoset, we found only one ERV sequence near *RHOXF2*, which is likely due to the insufficient coverage of this genomic region.

Compared with the human sequences (1/783), the orthologous nonhuman primate ERV sequences are highly diverged (20/780 in chimpanzee, 90/776 in gorilla, 57/767 in orangutan and 109/775 in rhesus macaque) (see additional file 5). This implies that frequent non-allelic recombinations might not have occurred in nonhuman primates involving two *RHOXF2 *copies (rhesus macaque, pig-tailed macaque, grey leaf and black leaf monkey). For example, in the rhesus macaque, we identified only two *RHOXF2 *coding-region haplotypes with six fixed between-copy substitutions (see additional file 2). This is consistent with previous computational analysis as well as data for *Arabidopsis*, which proposed that the recombination frequency decreases very rapidly with the increase of sequence divergence [33,34]. In chimpanzee, there are more than two ERV sequences (figure 1b), resulting in a more complicated pattern requiring further illumination through future detailed sequence analysis.

Additionally, for the 11 primate species with no heterozygous site, the distinct sequence divergence between the flanking ERVs (in gorilla and orangutan) is consistent with our proposal of one *RHOXF2 *copy in these species, as determined by genomic DNA qPCR (figure 3).

### Multi-lineage positive selection on primates

We conducted coding sequence comparison among the primate species as well as phylogenetic-tree-based analysis for the molecular signatures of selection. To simplify the phylogenetic analysis, we generated two sequences by randomizing the SNPs of humans and chimpanzees respectively to represent their RHOXF2 coding sequences. Different combinations of randomizing the SNPs gave rise to the same results, and it remains unaffected under the most conservative scenario in which the sequence was reconstructed in each species without any non-synonymous changes (see additional file 6). All of the sequences from the other primates have distinctive haplotype sequences confirmed by clone sequencing. The intact copy of the marmoset sequence was used as an out-group.

The aligned protein sequences revealed high substitution rates (as well as frequent in-dels) for *RHOXF2 *in Catarrhini primates (see additional file 7) We found that 69.8% (206/295) of sites have become variable since the most recent ancestor of Catarrhine. In other words, only 30.2% of amino acids are identical among the 17 primate species, an indication of rapid evolution. Besides amino acid changes, there are also multiple deletions/insertions of short amino acid fragments, especially along the lineages to humans and chimpanzees (figure 2). Notably, the fixed protein sequence divergence between human and chimpanzee is 5.7% (16/281), which is much higher than the genome average (1.34%) [35].

**Figure 4:**
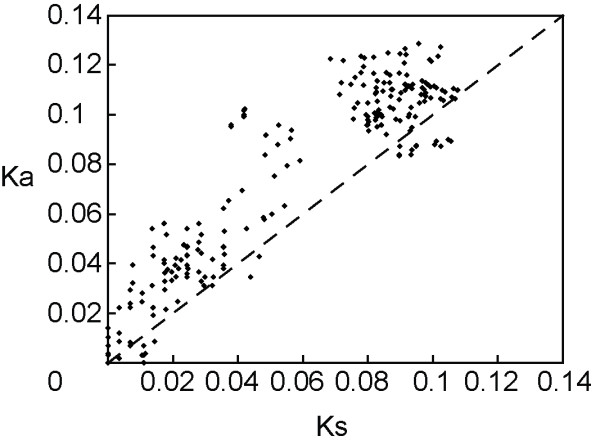
**Plot of pairwise Ka/Ks ratios in the primate species**. There are 222 out of the 253 pairwise Ka/Ks ratios (87.7%) showing large values (>1). Ka/Ks ratios were estimated following Pamilo-Bianchi-Li's method [65,66].

The comparison of non-synonymous (Ka) and synonymous (Ks) nucleotide distances between gene sequences can detect selection acting on a gene. A high Ka/Ks ratio (>1) indicates positive selection, whereas a lower Ka/Ks ratio indicates negative selection (<1). The Ka versus Ks ratios for all species indicated high values (Ka/Ks > 1) in 222 out of the 253 pairs (87.7%) (figure 4, see additional file 8), an implication of deviation from the expectation of neutrality (Ka/Ks = 1) and strong positive selection on *RHOXF2 *during primate evolution. The signal of positive selection in primates was confirmed by comparing *Model 2a *(selection) and *Model 1a *(neutral) using the maximum-likelihood method (2ΔLnL = 31.88, P = 0.000000194) [36]. We also examined the detailed substitution pattern of each primate lineage in the phylogenetic tree (figure 2). The Ka/Ks ratios of many primate lineages are larger than one, and some are statistically significant, especially the lineages leading to humans and chimpanzees, and the lineage to the Yunnan snub-nosed monkey (figure 2) [37,38].

Notably, all of the Ka/Ks ratios for the lineages leading to humans and chimpanzees since the most recent common ancestor of Catarrhines are larger than 1 (thick lines in figure 2). The likelihood ratio test (modified model A test) indicated that these lineages have a significantly larger ω (*d*N*/d*S) value (>1) (2ΔLnL = 4.23, P < 0.05), suggesting strong positive selection during the evolution of humans and chimpanzees (table 2). In comparison, another *Rhox *family member *RHOXF1*, located in the same genomic region (figure 1a), evolved much more slowly than *RHOXF2 *(table 2).

**Table 2:** Comparison of sequence substitution rates of *RHOXF1 *and *RHOXF2 *in primates

	Human lineage			Non-human primates lineage		
	
	lineage	N/S	Ka/Ks	lineage	N/S	Ka/Ks
	A-HUM	5/1	1.01	A-CHP	11/0	∞
	B-HUM	9/1	1.99	B-GOR	6/4	0.72
*RHOXF2*	C-HUM	22/3	2.60*	C-ORA	9/7	0.61
	D-HUM	27/5	2.18	D-SIA	19/6	1.59
	E-HUM	33/7	1.24	E-RM	22/11	0.96

	A-HUM	0/3	0	A-CHP	5/1	1.52
*RHOXF1*	B-HUM	1/3	0.10	B-GOR	5/3	0.50
	C-HUM	6/4	0.44	C-ORA	4/3	0.38
	E-HUM	14/5	0.83	E-RM	13/1	3.87

Note: A-E are the inferred ancestral nodes of the primate phylogenetic tree (figure 2). N/S indicates the number of non-synonymous substitutions and the number of synonymous substitutions of each lineage. *Ka/Ks > 1, P = 0.026 (Z test)

In the 111 human individuals tested, we observed eight sequence polymorphisms (SNPs) and all of them are non-synonymous substitutions (83A/T, 93N/D, 151R/C, 151R/H, 176L/F, 209Q/H, 235G/D and 286 P/L). Surprisingly, three of them (151R/C, 151R/H and 176L/F) are located in the homeodomain (table 3). No synonymous substitutions were observed in the entire coding region of *RHOXF2*, an indication of on-going positive selection on current human populations. In the chimpanzee lineage (node A to chimpanzee ancestor), there are 5 non-synonymous substitutions while a 7-aa deletion without any synonymous substitution was located in the homeodomain (figure 2). The Ka/Ks ratio is significantly larger than one (P < 0.01, one-tailed Z test) [37], again supporting the hypothesis of strong positive selection on the *RHOXF2 *homeodomain during the evolution of humans and chimpanzees.

**Table 3:** The distribution of amino acid variations of *RHOXF2 *in human populations

	Allele frequency				
	
polymorphic site	African (n = 32)	European (n = 21)	Melanesian (n = 10)	East Asian (n = 48)	Total (n = 111)
83 Ala→Thr	0.0179	0	0	0	0.0047
93 Asn→Asp	0.5	0.643	0.750	0.723	0.660
151 Arg→Cys	0.15	0.143	0.250	0.170	0.167
151 Arg→His	0.0333	0	0	0	0.0093
176 Leu→Phe	0	0	0	0.052	0.0096
209 Gln→His	0.161	0.177	0	0.022	0.078
235 Gly→Asp	0.887	0.972	0.778	0.696	0.808
286 Pro→Leu	0.0968	0	0.050	0	0.0333

Note: The ancestral alleles in humans were determined by comparing with the non-human primate species. The frequencies shown are the derived alleles.

In Old World monkeys, *RHOXF2 *were duplicated twice independently, one in the leaf monkey lineage and the other in the macaque lineage (in the common ancestor of rhesus macaques and pig-tailed macaques). Theoretically, it is also possible that there have been three copy loss events (the ancestor of DL and YGM, the RG lineage, and the ancestor of STM, AM and PDM) which can explain the observed pattern although it is less parsimonious than the proposed two independent duplications. Strong positive selection was detected in the Yunnan snub-nosed monkey lineage as well as in the lineage including rhesus and pig-tailed macaques (figure 2). Taken together, during the evolution of primates, along with parallel gene duplications and/or losses, positive selection has been acting on multiple primate lineages leading to the rapid protein sequence changes of *RHOXF2*.

### Expression pattern of *RHOXF2 *in primates

**Figure 5:**
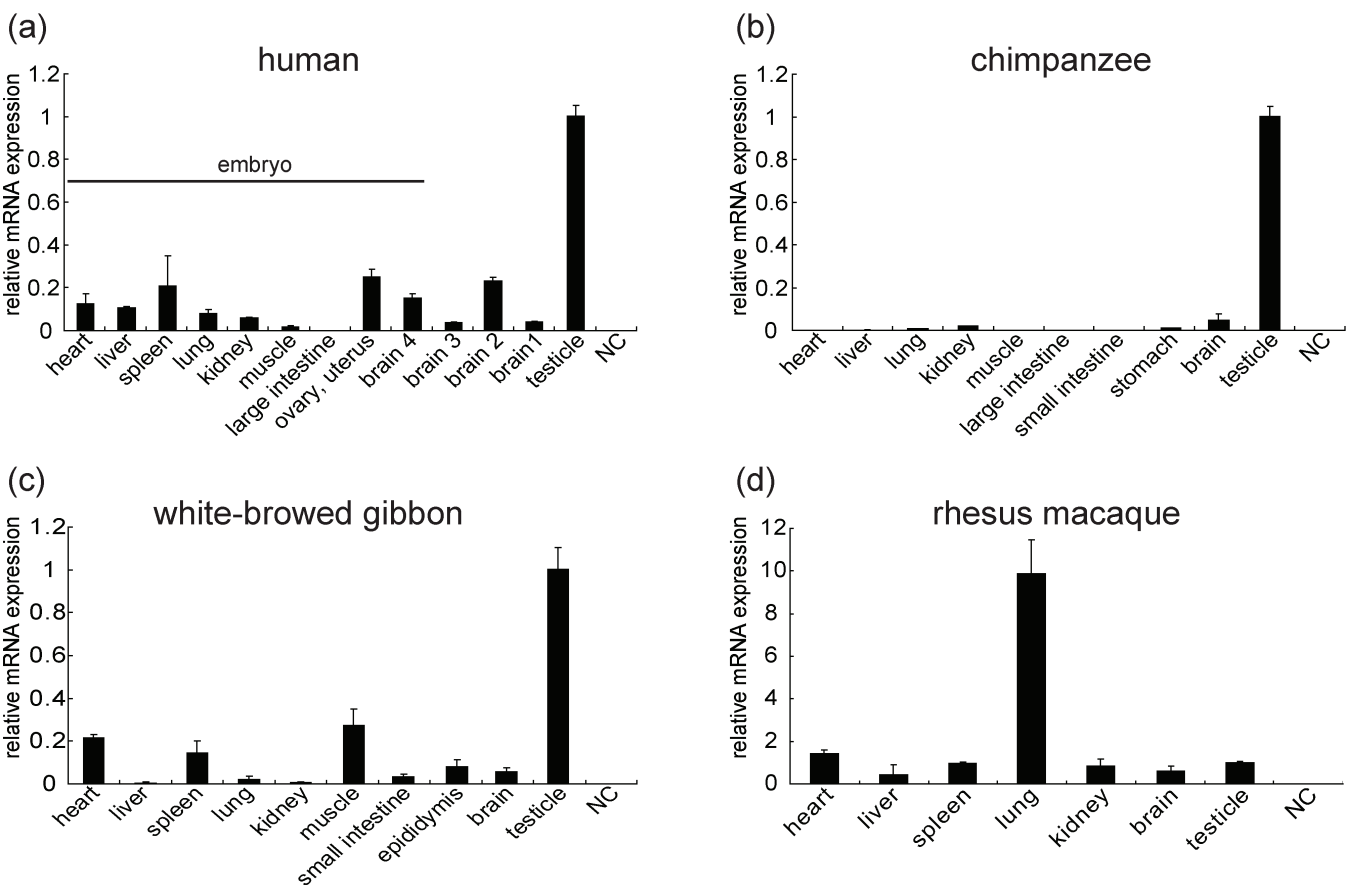
**The expression patterns of *RHOXF2 *determined by qPCR in humans(a), chimpanzee(b), white-browed gibbon(c) and rhesus macaque(d)**. The relative expression levels were calculated by setting the expression level in testicle as "1". For the human samples, Brain 1(40 yrs), Brain 2 (28 yrs), Brain 3 (newborn, 1 month) and testicle (76 yrs) are all male individuals. The human embryo is a 36-weeks female. The ages of the nonhuman primate samples are 2 yrs for the male chimpanzee, 6 yrs for the male white-browed gibbon and 20 yrs for the male rhesus macaque. "NC" refers to negative control. The detailed sample information of the nonhuman primate species is described in Materials and Methods.

To exam the expression pattern of *RHOXF2 *in primates, we performed real-time qPCR in four primate species (rhesus macaques, the white-browed gibbon, chimpanzee and humans). The general expression patterns in human, chimpanzee and gibbon are similar, consistent with the reported data in human and mice [15,39], in which *RHOXF2 *is preferentially expressed in the testis (figure 5). However, the rhesus macaque showed a very different expression pattern. *RHOXF2 *is expressed in all the major tissues, but the highest expression was observed in the lung instead of the testicle (figure 5). This was further confirmed by testing two more individuals (one 2 yr male and one 2 yr female) (see additional file 9). The functional implication of the preferential expression of *RHOXF2 *in the lung of rhesus macaque is yet to be dissected.

It is noteworthy that we also observed expression of *RHOXF2 *in the brain. In humans, *RHOXF2 *is weakly expressed in the brain compared with the testis, and there are between-individual and between-developmental-stage variations (figure 5). In Wayne *et al*. (2002), the brain expression of *RHOXF2 *was not detected, which was likely due to the insensitive technology (Northern blot) used. *RHOXF2 *is also expressed in the brains of chimpanzee, gibbon and rhesus macaque.

To examine the expression of individual *RHOXF2 *paralog, we cloned and sequenced cDNAs of the two *RHOXF2 *copies in humans, and screened 7 adult human testicle samples and 4 human brain samples. We found that both copies are almost equally expressed in all testicle samples (clone counting, 20:17; see additional file 3), implying that the two copies are functionally redundant in the human testis. However, in the human brain, the expression pattern is different. In the embryo and new born brains, both copies are expressed (clone counting, 34:20; see additional file 3), while only one copy is expressed in adult brains (clone counting, 35:0; see additional file 3), suggesting tissue and developmental stage related expression divergence of the two *RHOXF2 *copies during human evolution.

In the chimpanzee brain, we detected four cDNA sequences (clone counting, 34:45:4:1) and two of them are the major forms. One of the major forms (clone counting, 45) is a novel splice form that was not detected in the chimpanzee testis, however, this form produces a truncated protein. The other two minor forms likely represent background expression due to their truncated open-reading frames (see additional file 2, additional file 3) and low expression levels. Interestingly, all four brain-expressed forms are from only one of the six gene copies in chimpanzee, suggesting between-copy expression divergence, similar to the pattern observed in the human brain. In the rhesus macaque, the expression pattern is similar among testicle, brain and lung, in which Copy-1 is preferentially expressed (clone counting, 59:7 for testicle, 41:3 for brain and 19:0 for lung; see additional file 3).

## Discussion

We have presented a novel case of rapid evolution of an X-linked homeobox gene in primates. Interestingly, unlike the few previously studied cases [8-10,13,14], *RHOXF2 *also shows copy number variation among primate species. We showed that there had been parallel *RHOXF2 *duplications and/or losses along multiple primate lineages, which were likely mediated by the flanking ERVs. A similar gene duplication pattern was also observed for the mouse *Rhox *alpha subcluster paralogs [8-12], and gene loss was reported in the Hox gene cluster in nematode [5,6].

*RHOXF1 *and *RHOXF2 *are the only members of the *Rhox *family in primates [40]. Due to the rapid evolution of the *Rhox *family, *RHOXF1 *and *RHOXF2 *are highly divergent from their rodent orthologs. However, their functional roles in the reproductive system have been maintained in both rodents and primates. In the mouse, *Rhox5 *is expressed in both male and female germ cells in developing fetal gonad [41,42]. In adults, it is expressed in the testis, epididymis and ovary [43,44]. The *Rhox5 *null mice are subfertile, with defects in spermatozoa production and motility [8,41]. The mouse *Rhox *cluster has more than 30 genes with partially overlapping expression patterns, and plays partially redundant but distinct functional roles [8-10,18,45]. *RHOXF1 *and *RHOXF2 *in primates are functionally similar to *Rhox5 *in mouse. In the testis, both of them may down-regulate *Unc5c *and *Pltp *expression, but only *RHOXF2 *can up-regulate *Gdap1 *expression [18]. During primate evolution, *RHOXF1 *was apparently highly conserved and likely maintains its original functional role in the reproductive system. In contrast, *RHOXF2 *has evolved rapidly leading to potential functional divergence in primates.

Darwinian positive selection is likely the key driving force leading to copy number variation, rapid amino acid changes as well as potential functional (*e.g*. gene expression pattern) divergence of *RHOXF2 *in primates. In several species, *e.g*. rhesus macaque and leaf monkeys, selection and lack of recombination have caused distinct sequence divergence between the two *RHOXF2 *copies within each species, which could lead to functional divergence. In humans, signals of rapid evolution and positive selection were also detected. However, due to frequent non-allelic recombinations, the two human copies have not clearly diverged. The situation in chimpanzees is much more complicated in which both rapid evolution and gene pseudolization might have occurred.

It has been shown that gene copy number and expression level are highly correlated [46]. For homeobox genes, due to their important roles in development, dosage changes may lead to functional consequences [47,48]. As the major function of *RHOXF2 *in primates is male reproduction, we speculate that the observed strong selection in multiple primate lineages is likely to be related to sperm competition in promiscuous mating systems [49,50]. Among the 17 primate species studied, four species showed strong signatures of positive selection (human, chimpanzee, Yunnan snub-nosed monkey and rhesus macaque), and all of them have promiscuous mating systems [51-57]. This pattern is also seen for the *Rhox *cluster in rodents [8].

In addition to its potential role in spermatogenesis, *RHOXF2 *may also be involved in the functioning of the nervous system. All the three down-stream genes directly regulated by *RHOXF2*, *i.e. Unc5c *[19,20], *Pltp *[21,22] and *Gdap1 *[23,24] also play important roles in the central nervous system. Hence, the brain expression of *RHOXF2 *in primates implies its possible involvement in brain function. In humans, only one of the two *RHOXF2 *copies is expressed in adult brains, and both copies are expressed equally in the brains of embryos and new-borns. This suggests a potential role of *RHOXF2 *in brain development [16-18]. Thus, it is likely that *RHOXF2 *may function in both the male reproductive system and the central nervous system through interactions with the down-stream genes. The question remains as to just how the expression of *RHOXF2 *is regulated in these different tissues.

It has been shown that the genome-wide gene expression patterns are similar between brain and testis in humans [58]. Interestingly, the human *RHOXF2 *protein down-regulates the expression of the Netrin-1 receptor, *Unc5c *[16-18], expressed in both brain and testis [16,59]. In the brain, the *UNC5C *protein level is mainly influenced by the *Netrin-1 *protein, which increases apoptosis of the *UNC5C*-expressing neurons [60,61]. In the testis, *Unc5c *prevents Sertoli cell from apoptosis and ensure sperm production [16]. A genome-wide analysis of cancer-testis (CT) gene expression showed that *RHOXF2 *is a CT gene with testis-selective expression [39]. Coincidentally, the CT genes are also expressed in a high percentage of human central nervous system tumors [62]. The potential functional role of *RHOXF2 *in the brain is likely the outcome of Darwinian positive selection which has driven the rapid evolution and functional divergence of *RHOXF2 *in primates, especially those species with more than one gene copies.

It is well known that brain evolution is not always positively associated with reproductive fitness [63]. A mutation with advantages for brain function may sometimes be detrimental to the reproductive system (and vice versa). Therefore, natural selection will result in a balance between the competing demands and advantages of brain and testis functions. As *RHOXF2 *may play a dual role acting in the testis and brain, it may have developed certain mechanism for balancing potential functional conflicts between reproduction and cognition. One potential molecular mechanism is the between-copy gene expression divergence, *e.g*. in human, both of the *RHOXF2 *copies are equally expressed in the testis, but only one copy is expressed in the adult brain. The potential functional divergence of different copies are yet to be elucidated by further studies in the future.

## Conclusions

In summary, we provided an informative example of rapid evolution and copy number variation of an X-linked homeobox gene (*RHOXF2*) in primates. Our sequence analysis indicates that parallel gene duplications/losses were likely to have been mediated by the flanking ERVs, and the rapid evolution of *RHOXF2 *had been driven by Darwinian positive selection on the male reproductive system and possibly also on the central nervous system, resulting in between-copy sequence and expression divergence among the primate species with more than one gene copy.

## Methods

### Ethics statement

All of the DNA samples used in this study were taken from collections by the Kunming Cell Bank of CAS, Kunming Blood Center and Shanghai National Genome Center in China. The research protocol was approved by the internal review board of Kunming Institute of Zoology, Chinese Academy of Sciences.

### Human and non-human primate DNA samples

For DNA samples, a total of 111 human individuals from the major continental populations were sampled and sequenced, including 32 Africans, 21 Europeans, 10 Melanesians and 48 East Asians (Chinese and Cambodian). We also sampled 16 nonhuman primate species (10 Old World monkey species, 3 lesser ape species and 3 great ape species) reflecting a 25 million-year history of primate evolution (table 1). The sequences of a New World monkey species, white-tufted-ear marmoset (*Callithrix jacchus*) were obtained from the database (http://genome.wustl.edu).

### Genomic DNA PCR, cloning and sequencing

The coding region of the *RHOXF2 *gene (exon 1-4) was amplified by PCR and sequenced in humans and 16 nonhuman primate species. Universal primers for all species were designed based on published sequences of human and other primate species. The primer sequences are listed in additional file 10. We first sequenced the PCR products directly. Then the PCR products were cloned into a pMD19-T vector using the T-vector kit (Takara, Japan) and then transformed into *E. coli *DH5α. The individual clones were picked for sequencing. Sequencing was performed in both directions with the forward and reverse primers on an ABI-3130 automated sequencer.

### BAC library screening

The pooled PCR-based method was used to screen a chimpanzee BAC library (CHORI251). According to the published sequences, the PCR primers were designed for BAC library screening. The best primer pairs were selected from the designed PCR primers through a series of pre-experiments. Primer sequences are shown in additional file 10. The BAC end sequencing was performed for all the positive clones [64].

### RNA samples, reverse transcriptional PCR (RT PCR), cloning and sequencing

We collected various organs (heart, liver, spleen, lung, kidney, muscle, intestine, pancreas, testicle and brain *etc.*) of three Chinese rhesus macaques (*Macaca mulatta*) (one male and one female 2 years old, and one male 20 years old), one white-browed gibbon (*Hylobates hoolock*) (male, 6 years old) and one chimpanzees (*Pan troglodytes*) (male, 2 years old). We also collected human brain samples from one new born male (1 month) and two adult males (28 years, 40 years), and multiple tissue samples from a female embryo (36 weeks). cDNAs from 2 rhesus macaque testis, 7 human testis, 2 chimpanzee testis were used for full length cDNA sequencing.

The total RNA samples were extracted with TRIzol (Invitrogen, Carlsbad, CA) following a standard protocol. They were treated with DNaseI (Takara, Tokyo, Japan) to remove possible genomic DNA contamination, then subject to reverse transcription using Omniscript Reverse Transcriptase (Qiagen, Valencia, CA) with oligo-dT primers (18 nucleotides), following the manufacturer's protocol. PCR was carried out at 95°C for 5 min, and then at 95°C for 30s, 59°C for 30s, and 72°C for 30s (or 2 min) for 35 cycles, and finally 72°C for 10 min. The primer sequences are presented in additional file 10. Cloning and sequencing were performed as described for the genomic DNA PCR products.

### Gene copy number and expression level determination by real-time quantitative PCR

The quantitative real-time PCR (qPCR) was performed using the SYBR premix ExTaq II (Takara, Tokyo, Japan) on a LightCycler 480 (Roche, Basel, Switzerland). For gene copy number estimation, the relative quantification method based on ΔΔCt was used to determine the relative copy numbers of the *RHOXF2 *gene in different species by comparison with a known single copy X-linked gene, transketolase-like 1 (*TKTL1*). The amplification efficiencies of *RHOXF2 *and *TKTL1 *in human and other non-human primates were tested and proven to be equal. PCR was carried out at 95°C for 4 min, and then at 95°C for 20s, 61°C for 20s for 40 cycles. For humans, chimpanzees, gorillas, orangutans and gibbons, the primers sequences are: NM032498_RT_F AGGGCATCAATGGCAAGAAAC and NM032498_RT_R AGGCTGCTGGAATGGCTGT; NM012253_RT_F TGGCAATCTTTGATGTGAACCG and NM012253_RT_R GGGGCAGGACAGAATGGAAAT. For the Old World monkeys, the primer pairs are: PEPP2-RT-owm-F AGAAGAGCCAAGTGGAGGAGACA and PEPP2-RT-owm-R GCAGTTACCATGACAGGCTGG; TKTL1-RT-owm-F CTACCGGGTGTTCTGCCTCAT and TKTL1-RT-owm-R AGATTGTCCAGACTGTAGTAGGAAGCA.

For measuring the gene expression levels, cDNA real-time PCRs were performed and the expression levels were determined by using glycerol-3-phosphate dehydrogenase (GAPDH) as the internal reference gene. PCR was carried out at 95°C for 2 min, and then at 95°C for 10s, and 64°C for 20s for 40 cycles. A variety of tissue types (heart, liver, spleen, lung, kidney, muscle, intestine, pancreas, testicle and brain *etc.*) were tested in Chinese rhesus macaques (one male and one female of 2 years old, and one male of 20 years old), white-browed gibbon (one 6 yrs male), chimpanzee (one 2 yrs male) and human (three brain samples, one testicle sample and multiple tissue samples from one female embryo). The GAPDH primers are identical in all four primate species: GAPDH_F ATTGCCCTCAACGACCACTTT and GAPDH_R GGTCTCTCTCTTCCTCTTGTGCTCT. The *RHOXF2 *primer pairs are: for humans, pepp2-HUM-QF1 CGTCCACGCCTTCACCCC and pepp2-HUM-QR1 GTCTCCTCCATTTGGCTCTTCTATT; for chimpanzees, PEPP2-cRT-chp-hum-F1 CGAGCAGTTCCCCAGTGAGTT and PEPP2-cRT-chp-R1 CCATTGATGCCCTCTGATGTCTC; for the white-browed gibbon, pepp2-GB-QF3 ACTACAGGATATGAATGCTGCGGT and pepp2-GB-QR3 TGCTGCTTCTGTGCCTTGCT; For rhesus macaques, pepp2-RM-QF2 CAGGAGCTGGAGCGCATTTTC and pepp2-RM-QR2 CCTCCACTTGGCTCTTCTATTCTCA. The PCR product lengths are 164bp, 128bp, 100bp and 132bp respectively. The primer pairs are from different exons of *RHOXF2 *to avoid potential genomic DNA contamination. The list of the primers used is given in additional file 10. For each tissue sample, one RNA extraction was prepared, and the qPCR was repeated three times.

### Fluorescence *in situ *hybridization (FISH)

The positive BAC clone DNA was extracted according to the modified alkaline-lysis method. CHORI251-281C1 BAC clone DNA was labeled by Nick-translation with Biotin-dCTP (Invitrogen). The FISH method was described previously [64]. To eliminate the effect of cross-hybridization of common repeat sequences, the probes were blocked by using repetitive DNA (Cot) before hybridization. At least 10 independent metaphases or interphase nuclei were examined in determination of chromosomal band location.

### Data analysis

Sequence data was edited and aligned using *DNASTAR *(*DNASTAR, Inc.*), then inspected and confirmed manually. The DNA haplotypes (or copies) of humans were inferred using *PHASE *[25]. The haplotypes of other nonhuman primates were determined by cloning and sequencing when more than two heterozygous sites were observed. For phylogenetic analysis, a neighbor joining (NJ) tree was constructed using *MEGA4 *[38]. Synonymous and non-synonymous substitution rates (*Ks *and *Ka*) were calculated using Pamilo-Bianchi-Li's method [65,66], and the one-tailed *Z *test was used to detect deviation from neutrality [37,38]. Using *PAML *[36], the ancestral sequences of the internal nodes of the phylogenetic tree were inferred, and the substitution patterns were compared among different evolutionary lineages. Based on Yang's method [36], the selection model 2a (comparing with the neutral model 1a) was used to test positive selection in primates. Modified model A (thick lines in figure 2, *i.e*. the branches from the most recent common ancestor of Catarrhines to the human and chimpanzee lineages were taken as the foreground and compared with the corresponding null model with ω2 = 1 fixed) was used to test if there have been positive selections (Ka/Ks > 1) during the origins of humans and chimpanzees.

## Authors' contributions

BS, YQW and ALN conceived and designed the experiments. ALN, YQW, JC, CHL, JKW, RZ and HZ performed the experiments. YQW and ALN analyzed the data. BS, YQW and ALN wrote the paper. All authors read and approved the final manuscript.

## Note

[**GenBank**: HQ283451- HQ283472]

## Supplementary Material

Additional file 1 **Table S1 The relative *RHOXF2 *gene copies determined by qPCR in human population**. Using *TKTL1*, an X-linked single copy gene as control, the copy numbers were calculated by setting one male individual with heterozygous sites (**PG1302**) as "2". There are no *RHOXF2 *genomic DNA quantity difference between the group without heterozygous sites and the group with heterozygous sites (P = 0.12, T test). Click here for file

Additional file 2 **Figure S1 The coding DNA sequence alignment of primate *RHOXF2***. The coding DNA sequences of chimpanzee are denoted by schematic figures. Rhesus macaque (RM) CDS was inferred from cDNA; Pig-tailed macaque (PTM), black leaf monkey (BLM) and grey leaf monkey (GLM) CDSs were inferred from clone sequencing of genomic DNA. '.' indicates identical to the first sequence in each alignment. Click here for file

Additional file 3 **Table S2 The cDNA clone counting of *RHOXF2***. The brain and testicle samples of human, chimpanzee and rhesus macaque were included. The lung sample of rhesus macaque was also counted. Brain 1(40 yrs), brain 2 (28 yrs) and brain 3 (newborn, 1 month) are all male individuals. Brain 4 was from a 36 weeks female embryo. Click here for file

Additional file 4 **Figure S2 FISH (Fluorescence *In Situ *Hybridization) analysis in human, chimpanzee and gorilla using the *RHOXF2 *probe**. The red arrows indicate the positive signals. Click here for file

Additional file 5 **Figure S3 The ERV sequence alignment in human (hum), chimpanzee (CHP), gorilla (GOR), orangutan (ORA) and rhesus macaque (RM)**. '.' indicates identical to the first sequence in each alignment. '-' indicates an alignment gap. Click here for file

Additional file 6 **Figure S4. The phylogenetic tree showing sequence substitution pattern of each primate lineage**. The numbers of non-synonymous and synonymous substitutions (N/S) and the amino-acid insertions-deletions are labeled for each lineage. The sequences of of human and chimpanzee were reconstructed by excluding the within-species non-synonymous changes. Using marmoset sequence as outgroup, all internal node sequences were inferred by PAML. The lineages showing Ka/Ks ratios significantly larger than one are denoted by '*' (p < 0.05) or '**' (p < 0.01). For the abbreviations of the primate species, refer to table 1. Click here for file

Additional file 7 **Figure S5 Protein sequence alignment of primate *RHOXF2***. '.' indicates identical to the first sequence in each alignment. '-' indicates an alignment gap and '*' indicates a stop codon. The homeodomain region and proline-rich domain region are underlined. HUM-1/HUM-2 and CHP-1/CHP-2 represent the polymorphic sites in populations and the sequence difference among the within-species copies. The ancestral amino acid 151R is still in human population besides 151H and 151C. For the abbreviations of the primate species names, refer to table 1. Click here for file

Additional file 8 **Table S3 The pairwise Ka/Ks ratios of *RHOXF2 *in the primate species tested**. The Ka/Ks ratios were estimated following Pamilo-Bianchi-Li's method. Click here for file

Additional file 9 **Figure S6 The expression pattern of *RHOXF2 *determined by qPCR in two extra rhesus macaque individuals**. (a) a 2 yr male; (b) a 2 yr female. The relative expression levels were calculated by setting the value in the testicle as "1". The result is consistent with the data presented in Figure 5. Click here for file

Additional file 10 **Table S4 The primer sequences of PCR and sequencing in this study**. Click here for file

## References

[B1] Gehring WJ Affolter M Burglin T Homeodomain proteins Annu Rev Biochem 1994 63 487 526 10.1146/annurev.bi.63.070194.002415 7979246

[B2] McGinnis W Hart CP Gehring WJ Ruddle FH Molecular cloning and chromosome mapping of a mouse DNA sequence homologous to homeotic genes of Drosophila Cell 1984 38 3 675 680 10.1016/0092-8674(84)90262-9 6091896

[B3] Zhang J Nei M Evolution of Antennapedia-class homeobox genes Genetics 1996 142 1 295 303 8770606 10.1093/genetics/142.1.295 PMC1206958

[B4] Ting CT Tsaur SC Wu ML Wu CI A rapidly evolving homeobox at the site of a hybrid sterility gene Science 1998 282 5393 1501 1504 9822383 10.1126/science.282.5393.1501

[B5] Aboobaker AA Blaxter ML Hox Gene Loss during Dynamic Evolution of the Nematode Cluster Curr Biol 2003 13 1 37 40 10.1016/S0960-9822(02)01399-4 12526742

[B6] Aboobaker A Blaxter M Hox gene evolution in nematodes: novelty conserved Curr Opin Genet Dev 2003 13 6 593 598 10.1016/j.gde.2003.10.009 14638320

[B7] Sutton KA Wilkinson MF Rapid evolution of a homeodomain: evidence for positive selection J Mol Evol 1997 45 6 579 588 10.1007/PL00006262 9419235

[B8] Maclean JA Chen MA Wayne CM Bruce SR Rao M Meistrich ML Macleod C Wilkinson MF Rhox: a new homeobox gene cluster Cell 2005 120 3 369 382 10.1016/j.cell.2004.12.022 15707895

[B9] MacLean JA Lorenzetti D Hu Z Salerno WJ Miller J Wilkinson MF Rhox homeobox gene cluster: recent duplication of three family members Genesis 2006 44 3 122 129 10.1002/gene.20193 16496311

[B10] Wang X Zhang J Remarkable expansions of an X-linked reproductive homeobox gene cluster in rodent evolution Genomics 2006 88 1 34 43 10.1016/j.ygeno.2006.02.007 16574372

[B11] Morris L Gordon J Blackburn CC Identification of a tandem duplicated array in the Rhox alpha locus on mouse chromosome X Mamm Genome 2006 17 2 178 187 10.1007/s00335-005-0138-4 16465597

[B12] Jackson M Watt AJ Gautier P Gilchrist D Driehaus J Graham GJ Keebler J Prugnolle F Awadalla P Forrester LM A murine specific expansion of the Rhox cluster involved in embryonic stem cell biology is under natural selection BMC Genomics 2006 7 212 10.1186/1471-2164-7-212 16916441 PMC1562416

[B13] Wang X Zhang J Rapid evolution of mammalian X-linked testis-expressed homeobox genes Genetics 2004 167 2 879 888 10.1534/genetics.103.025072 15238536 PMC1470886

[B14] Wang X Zhang J Rapid evolution of primate ESX1, an X-linked placenta- and testis-expressed homeobox gene Hum Mol Genet 2007 16 17 2053 2060 10.1093/hmg/ddm153 17588961

[B15] Wayne CM MacLean JA Cornwall G Wilkinson MF Two novel human X-linked homeobox genes, hPEPP1 and hPEPP2, selectively expressed in the testis Gene 2002 301 1-2 1 11 10.1016/S0378-1119(02)01087-9 12490318

[B16] Hu Z Shanker S MacLean JA Ackerman SL Wilkinson MF The RHOX5 homeodomain protein mediates transcriptional repression of the netrin-1 receptor gene Unc5c J Biol Chem 2008 283 7 3866 3876 18077458 10.1074/jbc.M706717200

[B17] Hu Z MacLean JA Bhardwaj A Wilkinson MF Regulation and function of the Rhox5 homeobox gene Ann N Y Acad Sci 2007 1120 72 83 10.1196/annals.1411.011 18184911

[B18] Hu Z Dandekar D O'Shaughnessy PJ De Gendt K Verhoeven G Wilkinson MF Androgen-induced Rhox homeobox genes modulate the expression of AR-regulated genes Mol Endocrinol 2010 24 1 60 75 10.1210/me.2009-0303 19901196 PMC2802895

[B19] Round J Stein E Netrin signaling leading to directed growth cone steering Curr Opin Neurobiol 2007 17 1 15 21 10.1016/j.conb.2007.01.003 17254765

[B20] Ackerman SL Kozak LP Przyborski SA Rund LA Boyer BB Knowles BB The mouse rostral cerebellar malformation gene encodes an UNC-5-like protein Nature 1997 386 6627 838 842 10.1038/386838a0 9126743

[B21] Dong W Albers JJ Vuletic S Phospholipid transfer protein reduces phosphorylation of tau in human neuronal cells J Neurosci Res 2009 87 14 3176 3185 10.1002/jnr.22137 19472218 PMC2755571

[B22] Vuletic S Peskind ER Marcovina SM Quinn JF Cheung MC Kennedy H Kaye JA Jin LW Albers JJ Reduced CSF PLTP activity in Alzheimer's disease and other neurologic diseases; PLTP induces ApoE secretion in primary human astrocytes in vitro J Neurosci Res 2005 80 3 406 413 10.1002/jnr.20458 15795933

[B23] Liu H Nakagawa T Kanematsu T Uchida T Tsuji S Isolation of 10 differentially expressed cDNAs in differentiated Neuro2a cells induced through controlled expression of the GD3 synthase gene J Neurochem 1999 72 5 1781 1790 10217254 10.1046/j.1471-4159.1999.0721781.x

[B24] Cuesta A Pedrola L Sevilla T Garcia-Planells J Chumillas MJ Mayordomo F LeGuern E Marin I Vilchez JJ Palau F The gene encoding ganglioside-induced differentiation-associated protein 1 is mutated in axonal Charcot-Marie-Tooth type 4A disease Nat Genet 2002 30 1 22 25 10.1038/ng798 11743580

[B25] Stephens M Smith NJ Donnelly P A new statistical method for haplotype reconstruction from population data Am J Hum Genet 2001 68 4 978 989 10.1086/319501 11254454 PMC1275651

[B26] Andersson AC Yun Z Sperber GO Larsson E Blomberg J ERV3 and related sequences in humans: structure and RNA expression J Virol 2005 79 14 9270 9284 10.1128/JVI.79.14.9270-9284.2005 15994821 PMC1168766

[B27] Sun C Skaletsky H Rozen S Gromoll J Nieschlag E Oates R Page DC Deletion of azoospermia factor a (AZFa) region of human Y chromosome caused by recombination between HERV15 proviruses Hum Mol Genet 2000 9 15 2291 2296 11001932 10.1093/oxfordjournals.hmg.a018920

[B28] Bosch E Jobling MA Duplications of the AZFa region of the human Y chromosome are mediated by homologous recombination between HERVs and are compatible with male fertility Hum Mol Genet 2003 12 3 341 347 10.1093/hmg/ddg031 12554687

[B29] Kamp C Hirschmann P Voss H Huellen K Vogt PH Two long homologous retroviral sequence blocks in proximal Yq11 cause AZFa microdeletions as a result of intrachromosomal recombination events Hum Mol Genet 2000 9 17 2563 2572 10.1093/hmg/9.17.2563 11030762

[B30] Bosch E Hurles ME Navarro A Jobling MA Dynamics of a human interparalog gene conversion hotspot Genome Res 2004 14 5 835 844 10.1101/gr.2177404 15123583 PMC479110

[B31] Redon R Ishikawa S Fitch KR Feuk L Perry GH Andrews TD Fiegler H Shapero MH Carson AR Chen W Global variation in copy number in the human genome Nature 2006 444 7118 444 454 10.1038/nature05329 17122850 PMC2669898

[B32] Perry GH Ben-Dor A Tsalenko A Sampas N Rodriguez-Revenga L Tran CW Scheffer A Steinfeld I Tsang P Yamada NA The Fine-Scale and Complex Architecture of Human Copy-Number Variation The American Journal of Human Genetics 2008 82 3 685 695 10.1016/j.ajhg.2007.12.010 PMC2661628 18304495

[B33] Fujitani Y Kobayashi I Effect of DNA sequence divergence on homologous recombination as analyzed by a random-walk model Genetics 1999 153 4 1973 1988 10581300 10.1093/genetics/153.4.1973 PMC1460839

[B34] Opperman R Emmanuel E Levy AA The effect of sequence divergence on recombination between direct repeats in Arabidopsis Genetics 2004 168 4 2207 2215 10.1534/genetics.104.032896 15611187 PMC1448723

[B35] Chen FC Li WH Genomic divergences between humans and other hominoids and the effective population size of the common ancestor of humans and chimpanzees Am J Hum Genet 2001 68 2 444 456 10.1086/318206 11170892 PMC1235277

[B36] Yang Z PAML: a program package for phylogenetic analysis by maximum likelihood Comput Appl Biosci 1997 13 5 555 556 9367129 10.1093/bioinformatics/13.5.555

[B37] Kumar S Tamura K Jakobsen IB Nei M MEGA2: molecular evolutionary genetics analysis software Bioinformatics 2001 17 12 1244 1245 10.1093/bioinformatics/17.12.1244 11751241

[B38] Tamura K Dudley J Nei M Kumar S MEGA4: Molecular Evolutionary Genetics Analysis (MEGA) software version 4.0 Mol Biol Evol 2007 24 8 1596 1599 10.1093/molbev/msm092 17488738

[B39] Hofmann O Caballero OL Stevenson BJ Chen YT Cohen T Chua R Maher CA Panji S Schaefer U Kruger A Genome-wide analysis of cancer/testis gene expression Proc Natl Acad Sci USA 2008 105 51 20422 20427 10.1073/pnas.0810777105 19088187 PMC2603434

[B40] Holland PW Booth HA Bruford EA Classification and nomenclature of all human homeobox genes BMC Biol 2007 5 47 10.1186/1741-7007-5-47 17963489 PMC2211742

[B41] Pitman JL Lin TP Kleeman JE Erickson GF MacLeod CL Normal reproductive and macrophage function in Pem homeobox gene-deficient mice Dev Biol 1998 202 2 196 214 10.1006/dbio.1998.8978 9769172

[B42] Daggag H Svingen T Western PS van den Bergen JA McClive PJ Harley VR Koopman P Sinclair AH The rhox homeobox gene family shows sexually dimorphic and dynamic expression during mouse embryonic gonad development Biol Reprod 2008 79 3 468 474 10.1095/biolreprod.107.067348 18562707

[B43] Maiti S Doskow J Sutton K Nhim RP Lawlor DA Levan K Lindsey JS Wilkinson MF The Pem homeobox gene: rapid evolution of the homeodomain, X chromosomal localization, and expression in reproductive tissue Genomics 1996 34 3 304 316 10.1006/geno.1996.0291 8786129

[B44] Lindsey JS Wilkinson MF Pem: a testosterone- and LH-regulated homeobox gene expressed in mouse Sertoli cells and epididymis Dev Biol 1996 179 2 471 484 10.1006/dbio.1996.0276 8903361

[B45] MacLean JA Wilkinson MF The Rhox genes Reproduction 2010 10.1530/REP-10-0100 PMC7768892 20430877

[B46] Jung SH Shin SH Yim SH Choi HS Lee SH Chung YJ Integrated analysis of copy number alteration and RNA expression profiles of cancer using a high-resolution whole-genome oligonucleotide array Exp Mol Med 2009 41 7 462 470 10.3858/emm.2009.41.7.051 19322034 PMC2721143

[B47] Gage PJ Suh H Camper SA Dosage requirement of Pitx2 for development of multiple organs Development 1999 126 20 4643 4651 10498698 10.1242/dev.126.20.4643

[B48] Liu YH Tang Z Kundu RK Wu L Luo W Zhu D Sangiorgi F Snead ML Maxson RE Msx2 gene dosage influences the number of proliferative osteogenic cells in growth centers of the developing murine skull: a possible mechanism for MSX2-mediated craniosynostosis in humans Dev Biol 1999 205 2 260 274 10.1006/dbio.1998.9114 9917362

[B49] Anderson MJ Chapman SJ Videan EN Evans E Fritz J Stoinski TS Dixson AF Gagneux P Functional evidence for differences in sperm competition in humans and chimpanzees Am J Phys Anthropol 2007 134 2 274 280 10.1002/ajpa.20674 17632799

[B50] Muller MN Kahlenberg SM Emery Thompson M Wrangham RW Male coercion and the costs of promiscuous mating for female chimpanzees Proc Biol Sci 2007 274 1612 1009 1014 10.1098/rspb.2006.0206 17264062 PMC2141672

[B51] Ma SL Wang YX Jiang XL Li JX Study on the social behavior and habitual speciality of yunnan golden monkey Acta Theriologica Sinica 1989 9 3 161 167 (9)

[B52] Short RV Sexual selection and its component parts, somatic and genital selection, as illustrated by man and the great apes Adv Study Behav 1979 9 131 158

[B53] Harcourt AH Harvey PH Larson SG Short RV Testis weight, body weight and breeding system in primates Nature 1981 293 5827 55 57 10.1038/293055a0 7266658

[B54] Harcourt AH Purvis A Liles L Sperm competition: mating system, not breeding system, affects testes size of primates Funct Ecol 1995 9 468 476 10.2307/2390011

[B55] Dixson AF Anderson MJ Sexual behavior, reproductive physiology and sperm competition in male mammals Physiol Behav 2004 83 2 361 371 15488551 10.1016/j.physbeh.2004.08.022

[B56] Cui LW Huo S Zhong T Xiang ZF Xiao W Quan RC Social organization of black-and-white snub-nosed monkeys (Rhinopithecus bieti) at Deqin, China Am J Primatol 2008 70 2 169 174 10.1002/ajp.20471 17894403

[B57] Dixson AL Anderson MJ Sexual selection, seminal coagulation and copulatory plug formation in primates Folia Primatol (Basel) 2002 73 2-3 63 69 10.1159/000064784 12207054

[B58] Guo J Zhu P Wu C Yu L Zhao S Gu X In silico analysis indicates a similar gene expression pattern between human brain and testis Cytogenet Genome Res 2003 103 1-2 58 62 10.1159/000076290 15004465

[B59] Ackerman SL Knowles BB Cloning and mapping of the UNC5C gene to human chromosome 4q21-q23 Genomics 1998 52 2 205 208 10.1006/geno.1998.5425 9782087

[B60] Serafini T Colamarino SA Leonardo ED Wang H Beddington R Skarnes WC Tessier-Lavigne M Netrin-1 is required for commissural axon guidance in the developing vertebrate nervous system Cell 1996 87 6 1001 1014 10.1016/S0092-8674(00)81795-X 8978605

[B61] Yee KT Simon HH Tessier-Lavigne M O'Leary DM Extension of long leading processes and neuronal migration in the mammalian brain directed by the chemoattractant netrin-1 Neuron 1999 24 3 607 622 10.1016/S0896-6273(00)81116-2 10595513

[B62] Lee MH Son EI Kim E Kim IS Yim MB Kim SP Expression of cancer-testis genes in brain tumors J Korean Neurosurg Soc 2008 43 4 190 193 10.3340/jkns.2008.43.4.190 19096642 PMC2588259

[B63] Schillaci MA Primate mating systems and the evolution of neocortex size Journal of Mammalogy 2008 89 1 58 63 10.1644/06-MAMM-A-417.1

[B64] Xu HL Qian YP Nie WH Chi JX Yang FT Su B Construction, characterization and chromosomal mapping of bacterial artificial chromosome (BAC) library of Yunnan snub-nosed monkey (Rhinopithecus bieti) Chromosome Res 2004 12 3 251 262 15125639 10.1023/b:chro.0000021946.13556.40

[B65] Li WH Unbiased estimation of the rates of synonymous and nonsynonymous substitution J Mol Evol 1993 36 1 96 99 10.1007/BF02407308 8433381

[B66] Pamilo P Bianchi NO Evolution of the Zfx and Zfy genes: rates and interdependence between the genes Mol Biol Evol 1993 10 2 271 281 8487630 10.1093/oxfordjournals.molbev.a040003

