# Supplementary material for: Rapid evolution and copy number variation of primate RHOXF2, an X-linked homeobox gene involved in male reproduction and possibly brain function
Source: BMC Evol Biol. 2011 Oct 12;11:298. doi: 10.1186/1471-2148-11-298 (PMC3214919; doi:10.1186/1471-2148-11-298)
Supplement: Additional file 1 — Table S1 The relative RHOXF2 gene copies determined by qPCR in human population. Using TKTL1, an X-linked single copy gene as control, the copy numbers were calculated by setting one male individual with heterozygous sites (PG1302) as "2". There are no RHOXF2 genomic DNA quantity difference between the group without heterozygous sites and the group with heterozygous sites (P = 0.12, T test). [file 1471-2148-11-298-S1.DOC]

**Additional file 1.**

**Table S1 The relative *RHOXF2* gene copies determined by qPCR in human population.** Using *TKTL1*, an X-linked single copy gene as control, the copy numbers were calculated by setting one male individual with heterozygous sites (**PG1302**) as “2”. There are no *RHOXF2* genomic DNA quantity difference between the group without heterozygous sites and the group with heterozygous sites (P=0.12, T test).

|  | | △CT | △△CT | 2^－△△CT^ | Copy numbers |
| --- | --- | --- | --- | --- | --- |
| **Samples without heterozygous sites** | P205 | 0.2992 | -0.0176 | 1.0123 | 2.02 |
|  | P233 | 0.6099 | 0.2931 | 0.8161 | 1.63 |
|  | P234 | 0.2906 | -0.0262 | 1.0183 | 2.04 |
|  | P272 | 0.4153 | 0.0985 | 0.9340 | 1.87 |
|  | P280 | 0.6235 | 0.3067 | 0.8085 | 1.62 |
|  | ITA-33 | 0.4083 | 0.0915 | 0.9385 | 1.88 |
|  | PG1307 | 0.4309 | 0.1141 | 0.9240 | 1.85 |
|  | M30 | 0.0039 | -0.3129 | 1.2422 | 2.48 |
| **Samples having heterozygous sites** | ITA-7 | 0.0506 | -0.2662 | 1.2026 | 2.41 |
|  | PG1137 | -0.2388 | -0.5556 | 1.4698 | 2.94 |
|  | **PG1302** | **0.3168** | **0** | **1** | **2** |
|  | PG1304 | 0.5403 | 0.2235 | 0.8565 | 1.71 |
|  | PG1610 | -0.0786 | -0.3954 | 1.3153 | 2.63 |
|  | PG4020 | 0.5224 | 0.2056 | 0.8672 | 1.73 |
|  | M12 | 0.1572 | -0.1596 | 1.1170 | 2.23 |
|  | M17 | 0.3934 | 0.0766 | 0.9483 | 1.90 |
|  | M21 | 0.1252 | -0.1916 | 1.1420 | 2.28 |
