# Supplementary material for: Rapid evolution and copy number variation of primate RHOXF2, an X-linked homeobox gene involved in male reproduction and possibly brain function
Source: BMC Evol Biol. 2011 Oct 12;11:298. doi: 10.1186/1471-2148-11-298 (PMC3214919; doi:10.1186/1471-2148-11-298)

**Additional file 2.**

**Figure S1 The coding DNA sequence alignment of primate *RHOXF2*.** The coding DNA sequences of chimpanzee are denoted by schematic figures. Rhesus macaque (RM) CDS was inferred from cDNA; Pig-tailed macaque (PTM), black leaf monkey (BLM) and grey leaf monkey (GLM) CDSs were inferred from clone sequencing of genomic DNA. ‘.’ indicates identical to the first sequence in each alignment.


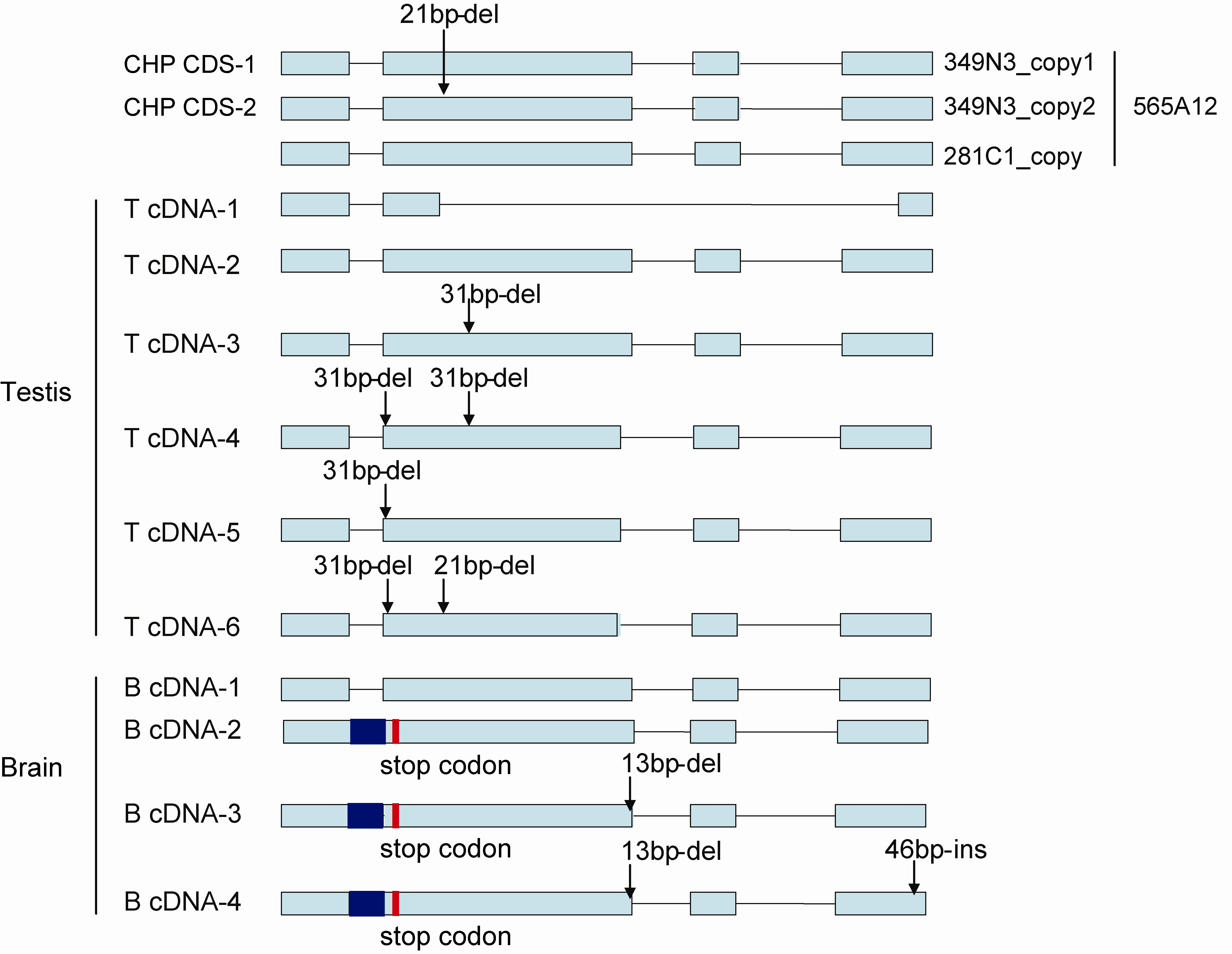


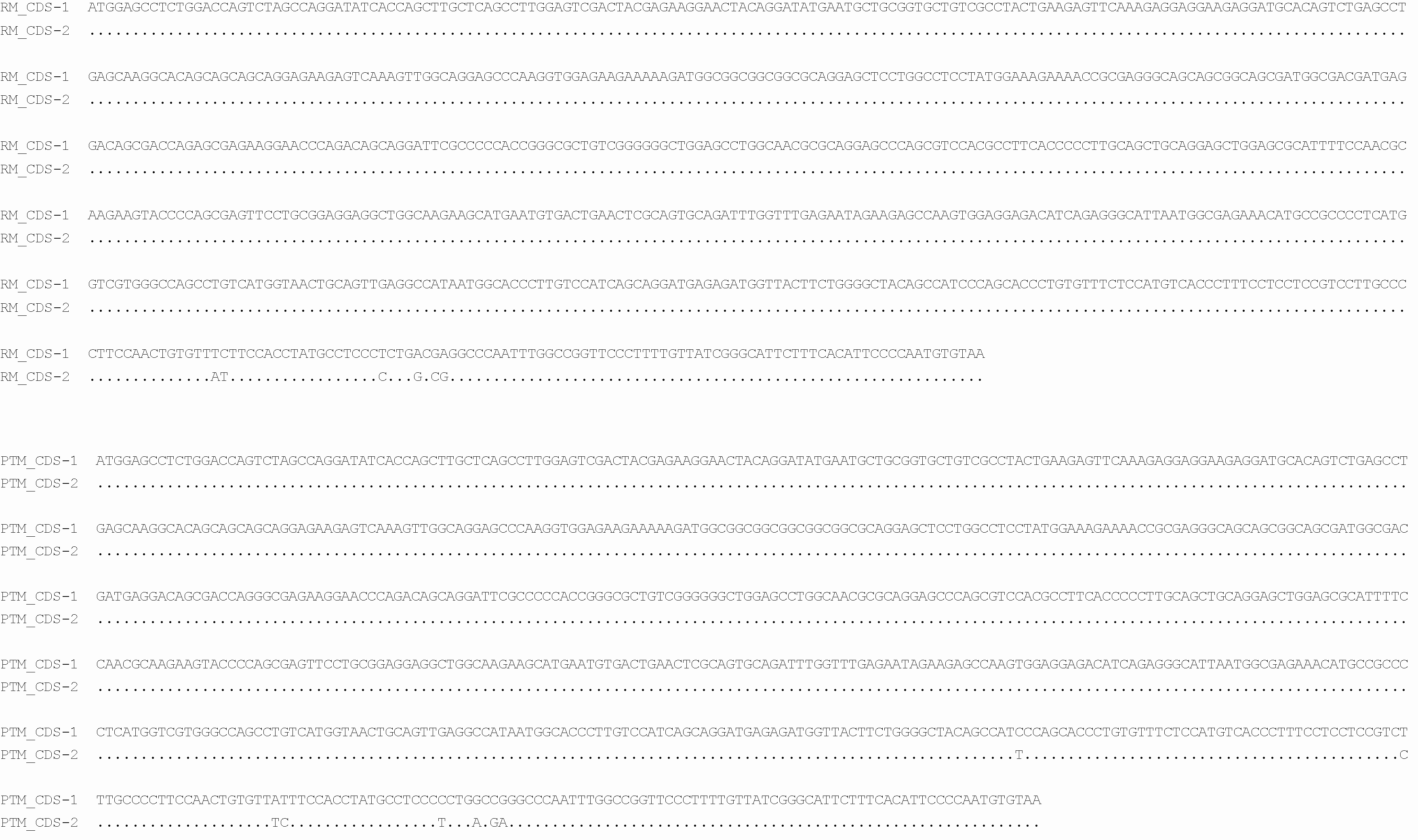


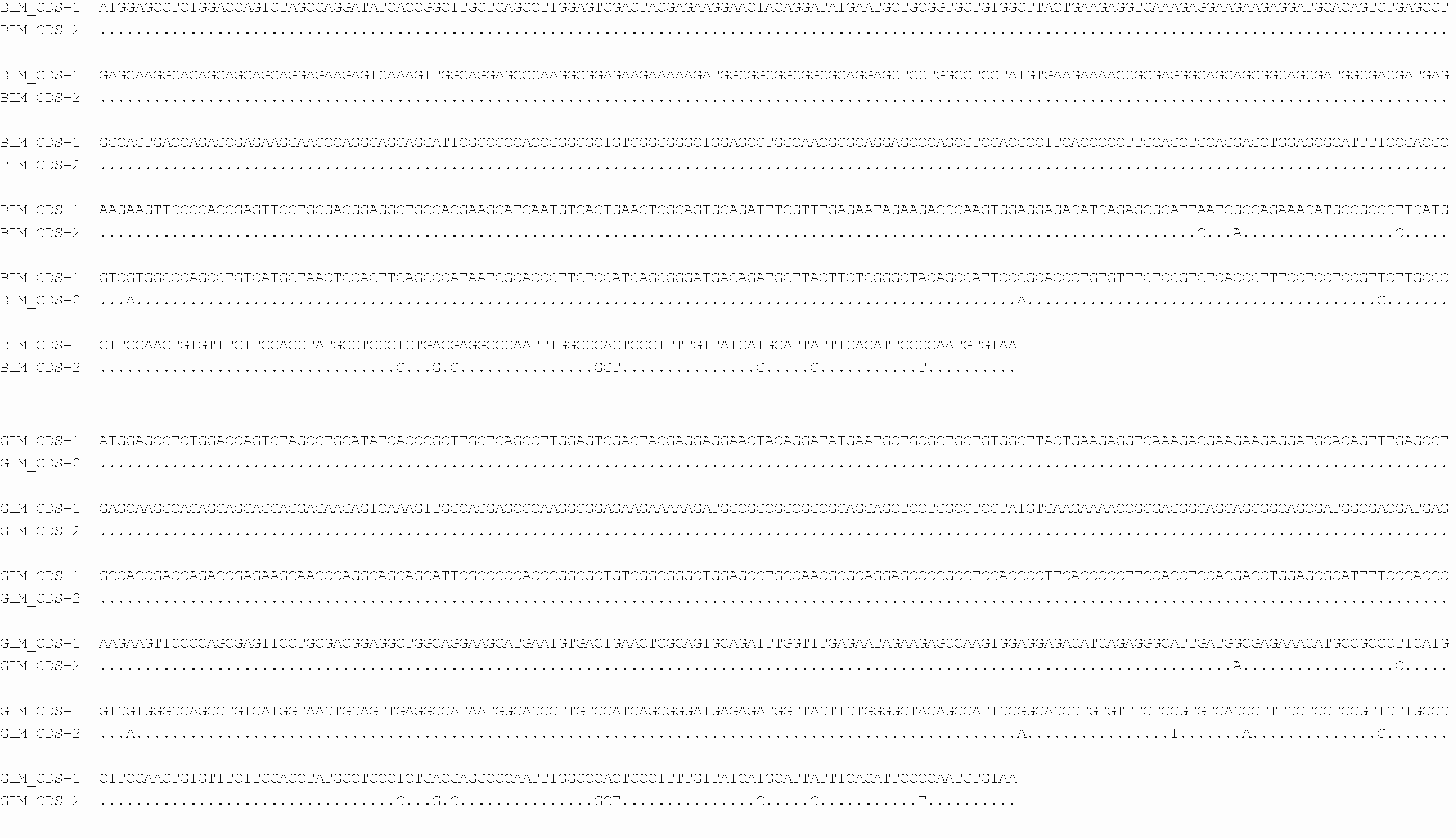

Supplement: Additional file 2 — Figure S1 The coding DNA sequence alignment of primate RHOXF2. The coding DNA sequences of chimpanzee are denoted by schematic figures. Rhesus macaque (RM) CDS was inferred from cDNA; Pig-tailed macaque (PTM), black leaf monkey (BLM) and grey leaf monkey (GLM) CDSs were inferred from clone sequencing of genomic DNA. '.' indicates identical to the first sequence in each alignment. [file 1471-2148-11-298-S2.DOC]
