# Supplementary material for: Rapid evolution and copy number variation of primate RHOXF2, an X-linked homeobox gene involved in male reproduction and possibly brain function
Source: BMC Evol Biol. 2011 Oct 12;11:298. doi: 10.1186/1471-2148-11-298 (PMC3214919; doi:10.1186/1471-2148-11-298)
Supplement: Additional file 3 — Table S2 The cDNA clone counting of RHOXF2. The brain and testicle samples of human, chimpanzee and rhesus macaque were included. The lung sample of rhesus macaque was also counted. Brain 1(40 yrs), brain 2 (28 yrs) and brain 3 (newborn, 1 month) are all male individuals. Brain 4 was from a 36 weeks female embryo. [file 1471-2148-11-298-S3.DOC]

**Additional file 3.**

**Table S2 The cDNA clone counting of *RHOXF2*. The brain and testis samples of human, chimpanzee and rhesus macaque were included.** The lung sample of rhesus macaque was also counted. Brain 1(40 yrs), brain 2 (28 yrs) and brain 3 (newborn, 1 month) are all male individuals. Brain 4 was from a 36 weeks female embryo.

|  | **Brain** | | **Testis** | | **lung** | |
| --- | --- | --- | --- | --- | --- | --- |
| **Human**  **(cds-1:cds-2)** | Brain1 | 23:0 | NJ1 | 5:5 | / | |
|  | Brain2 | 12:0 | NJ9 | 6:4 |  |  |
|  | Brain3 | 26:16 | NJ13 | 4:5 |  |  |
|  | Brain4 | 8:4 | Other(4) | 5:3 |  |  |
|  | total | 69:20 | total | 20:17 |  |  |
| **Chimpanzee**  **Brain (1:2:3:4)**  **Testis (1:2:3:4:5:6)** | Chp-A PFC | 6:25:1:0 | Chp-1 | 3:11:1:9:1:1 | / | |
|  | Chp-B IC | 26:0:0:0 | Chp-2 | 5:6:0:10:0:0 |  |  |
|  | Chp-B parietal lobe | 2:20:3:1 | / | / |  |  |
|  | total | 34:45:4:1 | total | 8:17:1:19:1:1 |  |  |
| **Rhesus macaque**  **(copy-1:copy-2)** | 86015 | 6:0 | 86015 | 4:0 | 86015 | 19:0 |
|  | 88087 | 41:3 | 88087 | 59:7 | / | / |
