# Supplementary material for: Rapid evolution and copy number variation of primate RHOXF2, an X-linked homeobox gene involved in male reproduction and possibly brain function
Source: BMC Evol Biol. 2011 Oct 12;11:298. doi: 10.1186/1471-2148-11-298 (PMC3214919; doi:10.1186/1471-2148-11-298)

**Additional file 4.**

**Figure S2 FISH (Fluorescence *In Situ* Hybridization) analysis in human, chimpanzee and gorilla using the *RHOXF2* probe.** The red arrows indicate the positive signals.


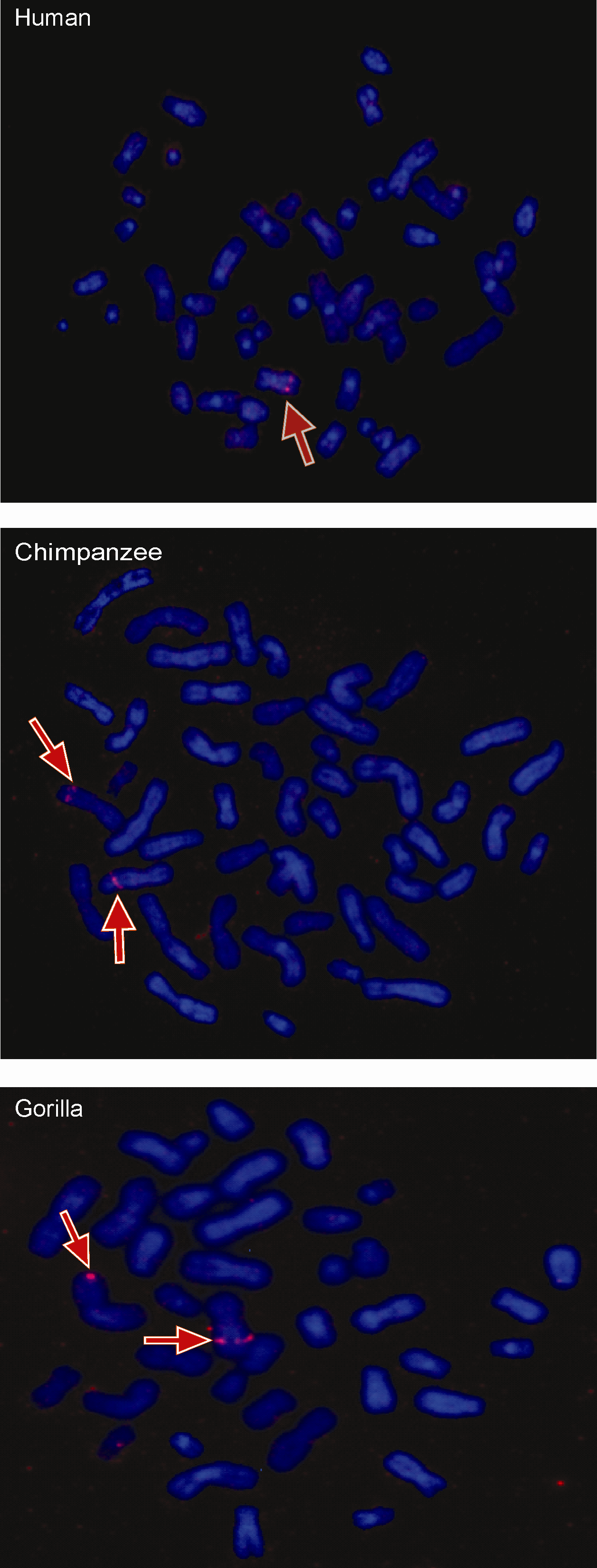

Supplement: Additional file 4 — Figure S2 FISH (Fluorescence In Situ Hybridization) analysis in human, chimpanzee and gorilla using the RHOXF2 probe. The red arrows indicate the positive signals. [file 1471-2148-11-298-S4.DOC]
