# Supplementary material for: Rapid evolution and copy number variation of primate RHOXF2, an X-linked homeobox gene involved in male reproduction and possibly brain function
Source: BMC Evol Biol. 2011 Oct 12;11:298. doi: 10.1186/1471-2148-11-298 (PMC3214919; doi:10.1186/1471-2148-11-298)

**Additional file 5. Figure S3 The ERV sequence alignment in human (hum), chimpanzee (CHP), gorilla (GOR), orangutan (ORA) and rhesus macaque (RM).** ‘.’ indicates identical to the first sequence in each alignment. ‘-’ indicates an alignment gap.


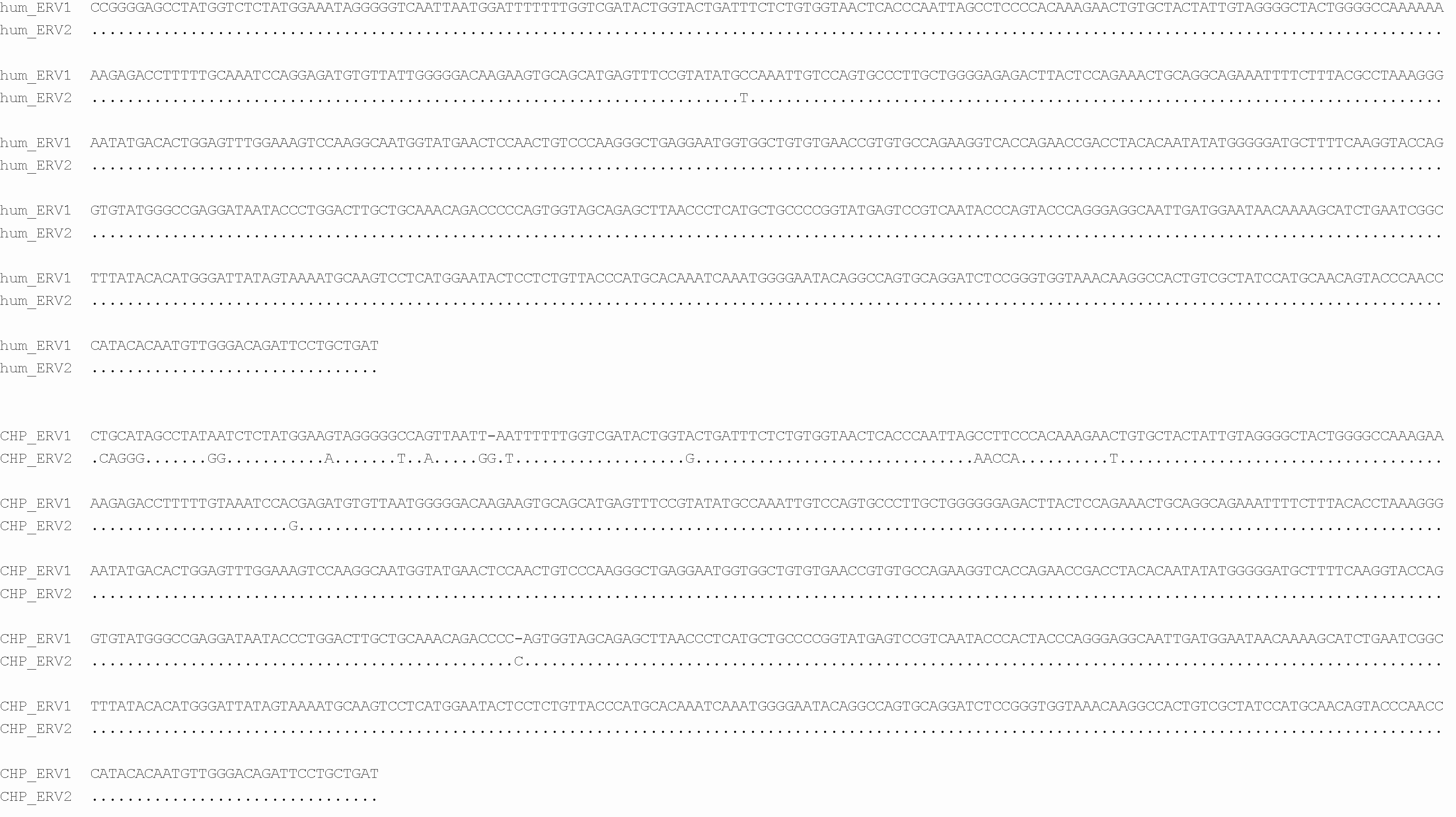


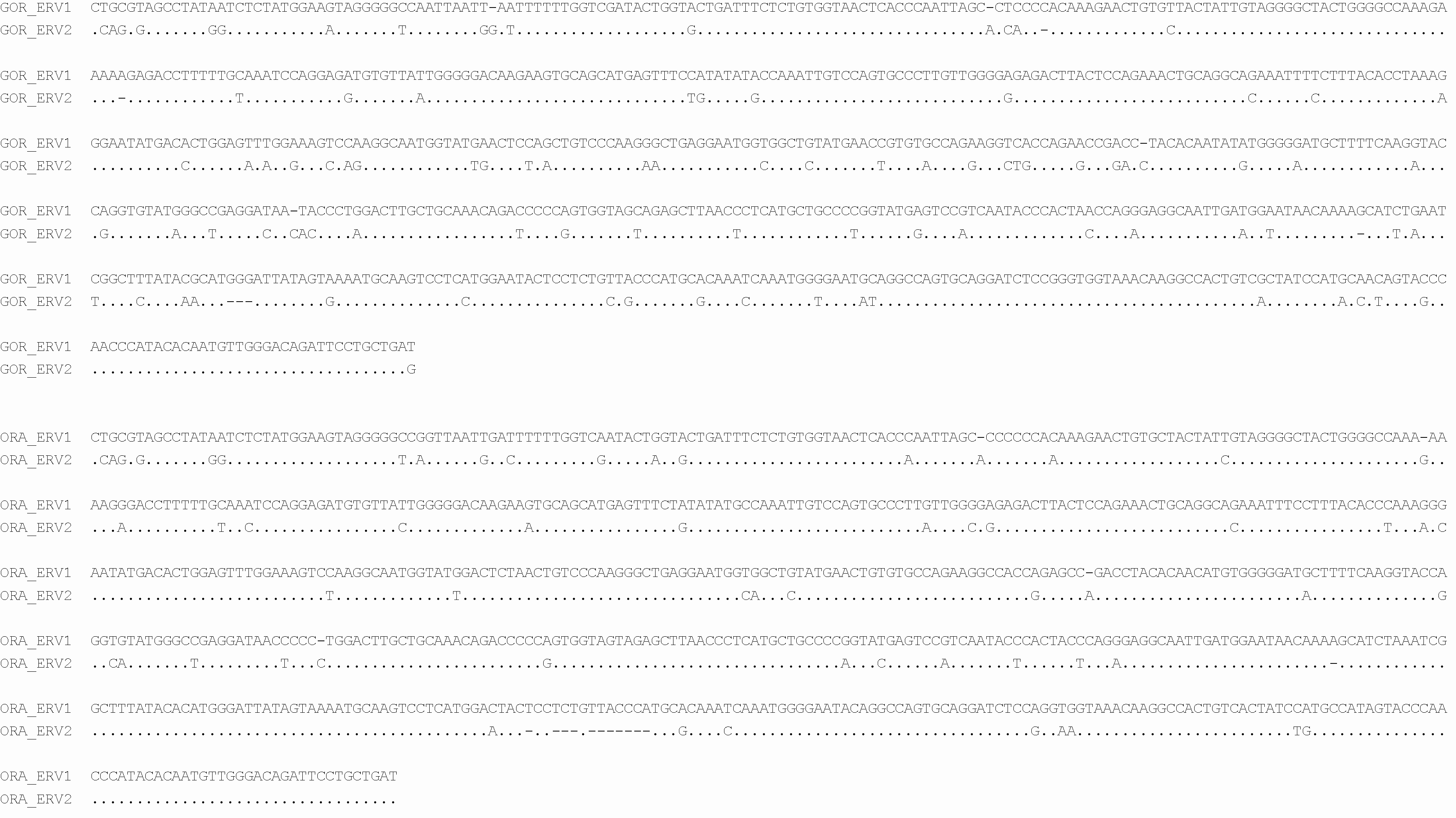


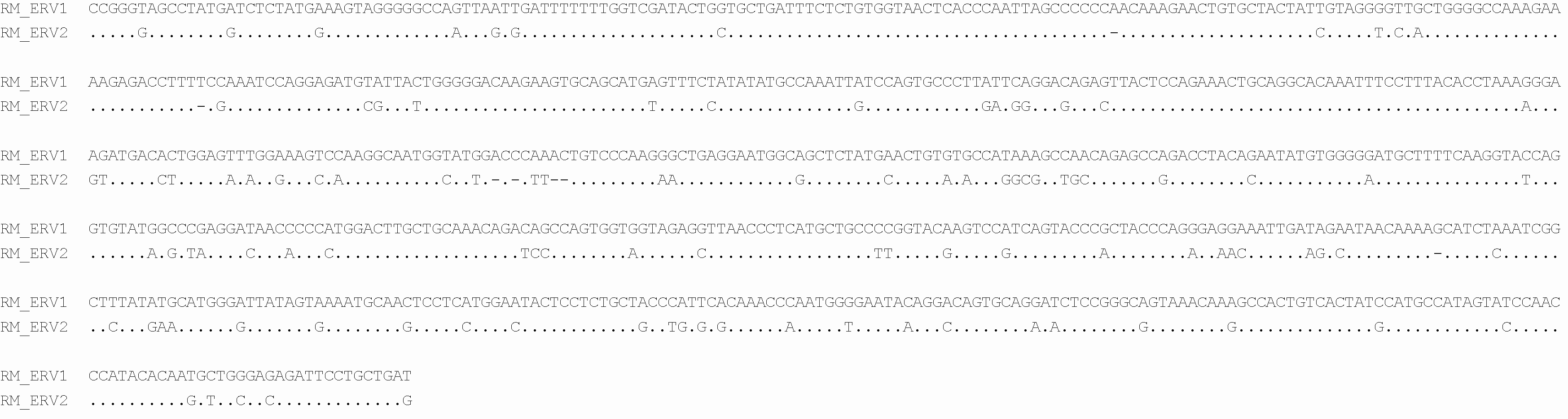

Supplement: Additional file 5 — Figure S3 The ERV sequence alignment in human (hum), chimpanzee (CHP), gorilla (GOR), orangutan (ORA) and rhesus macaque (RM). '.' indicates identical to the first sequence in each alignment. '-' indicates an alignment gap. [file 1471-2148-11-298-S5.DOC]
