# Supplementary material for: Rapid evolution and copy number variation of primate RHOXF2, an X-linked homeobox gene involved in male reproduction and possibly brain function
Source: BMC Evol Biol. 2011 Oct 12;11:298. doi: 10.1186/1471-2148-11-298 (PMC3214919; doi:10.1186/1471-2148-11-298)

**Additional file 6.**

**Figure S4. The phylogenetic tree showing sequence substitution pattern of each primate lineage.** The numbers of non-synonymous and synonymous substitutions (N/S) and the amino-acid insertions-deletions are labeled for each lineage. The sequences of of human and chimpanzee were reconstructed by excluding the within-species non-synonymous changes. Using marmoset sequence as outgroup, all internal node sequences were inferred by PAML. The lineages showing Ka /Ks ratios significantly larger than one are denoted by ‘*’ (p<0.05) or ‘**’ (p<0.01). For the abbreviations of the primate species, refer to table 1.


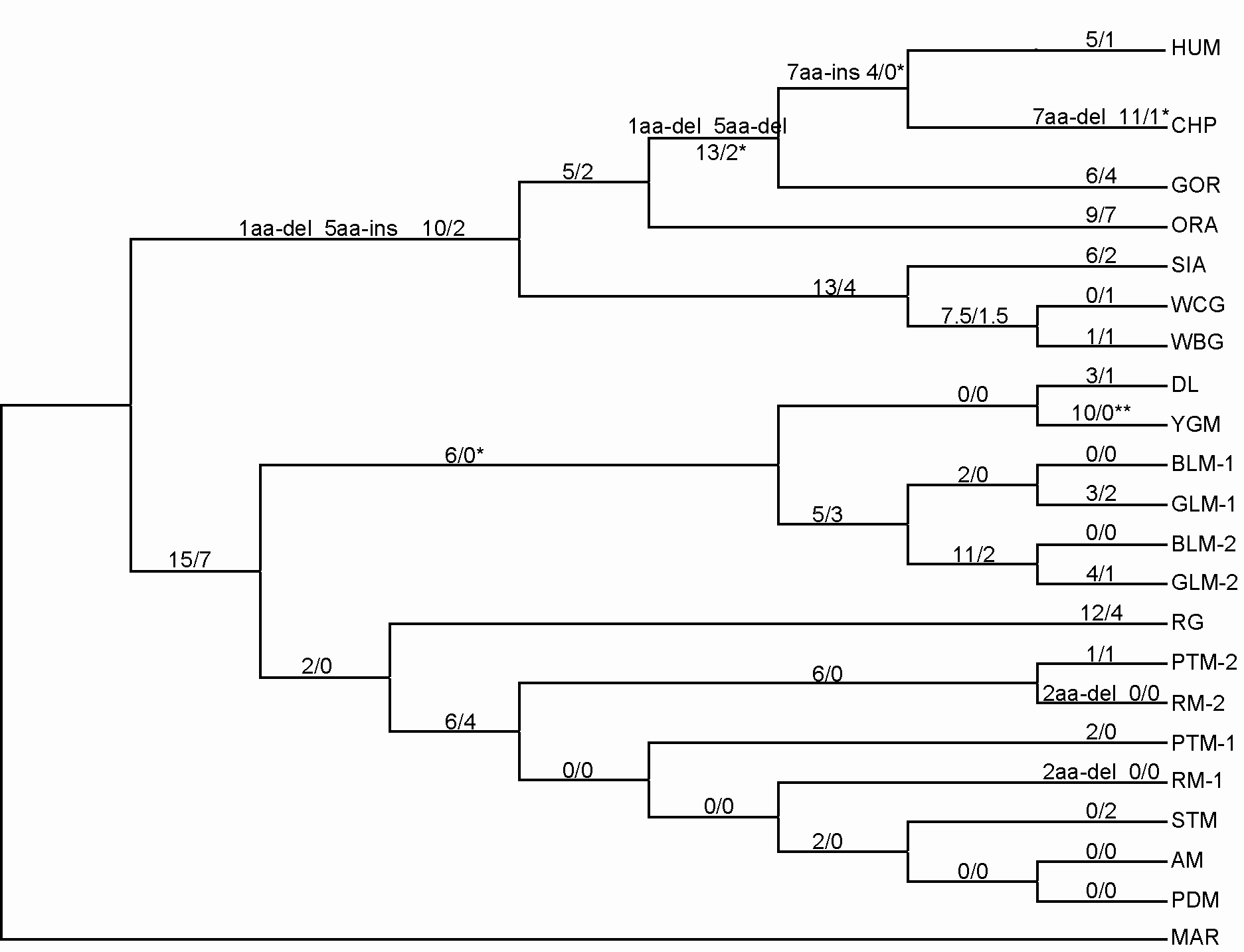

Supplement: Additional file 6 — Figure S4. The phylogenetic tree showing sequence substitution pattern of each primate lineage. The numbers of non-synonymous and synonymous substitutions (N/S) and the amino-acid insertions-deletions are labeled for each lineage. The sequences of of human and chimpanzee were reconstructed by excluding the within-species non-synonymous changes. Using marmoset sequence as outgroup, all internal node sequences were inferred by PAML. The lineages showing Ka/Ks ratios significantly larger than one are denoted by '*' (p < 0.05) or '**' (p < 0.01). For the abbreviations of the primate species, refer to table 1. [file 1471-2148-11-298-S6.DOC]
