# Supplementary material for: Rapid evolution and copy number variation of primate RHOXF2, an X-linked homeobox gene involved in male reproduction and possibly brain function
Source: BMC Evol Biol. 2011 Oct 12;11:298. doi: 10.1186/1471-2148-11-298 (PMC3214919; doi:10.1186/1471-2148-11-298)

**Additional file 7. Figure S5 The protein sequence alignment of primate *RHOXF2*.** ‘.’ indicates identical to the first sequence in each alignment. ‘-’ indicates an alignment gap and ‘*’ indicates a stop codon. The homeodomain region and proline-rich domain region are underlined. HUM-1/HUM-2 and CHP-1/CHP-2 represent the polymorphic sites in populations and the sequence difference among the within-species copies. The ancestral amino acide 151R is still in human population besides 151H and 151C. For the abbreviations of the primate species names, refer to table 1.


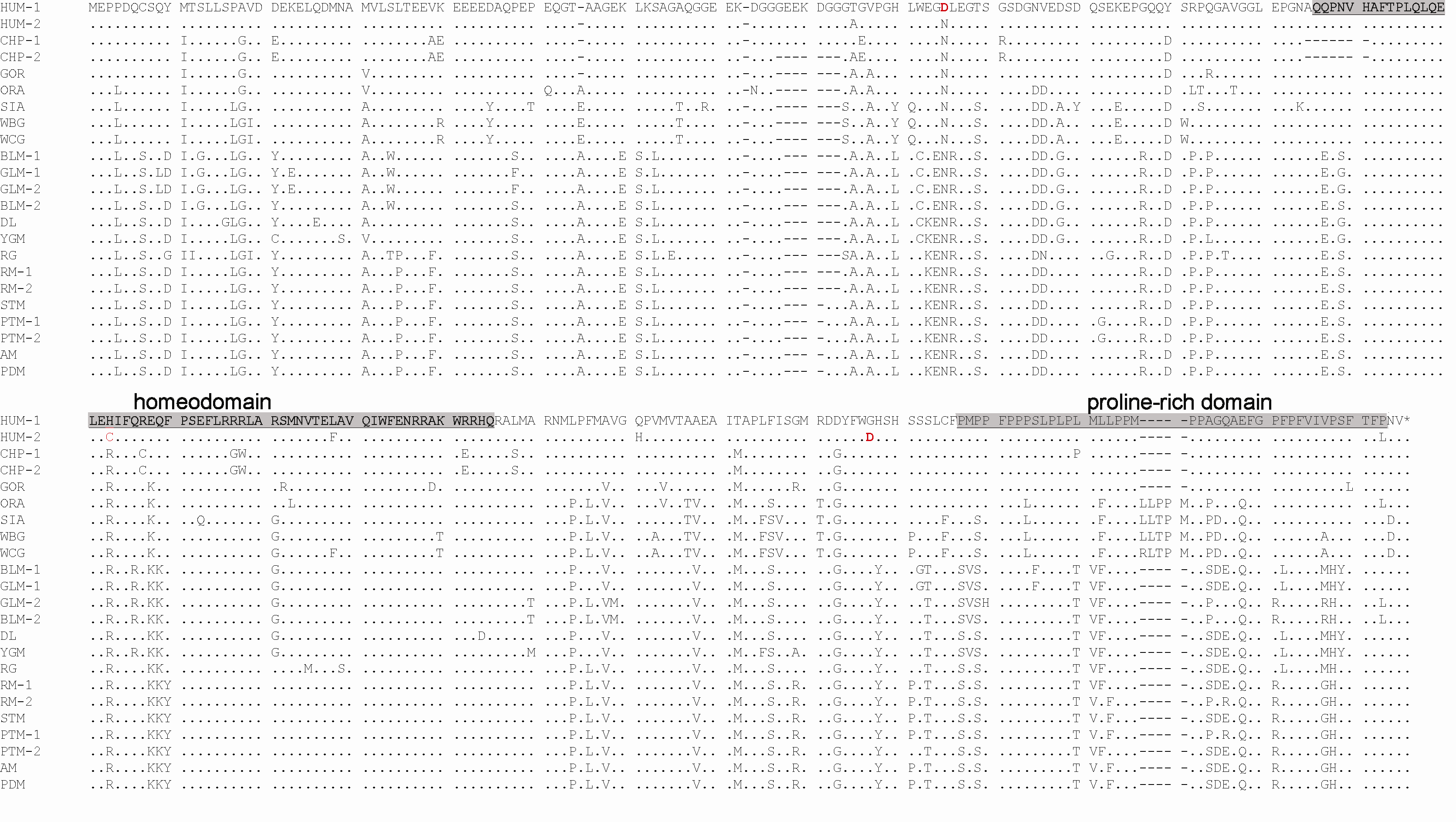

Supplement: Additional file 7 — Figure S5 Protein sequence alignment of primate RHOXF2. '.' indicates identical to the first sequence in each alignment. '-' indicates an alignment gap and '*' indicates a stop codon. The homeodomain region and proline-rich domain region are underlined. HUM-1/HUM-2 and CHP-1/CHP-2 represent the polymorphic sites in populations and the sequence difference among the within-species copies. The ancestral amino acid 151R is still in human population besides 151H and 151C. For the abbreviations of the primate species names, refer to table 1. [file 1471-2148-11-298-S7.DOC]
