# Supplementary material for: Rapid evolution and copy number variation of primate RHOXF2, an X-linked homeobox gene involved in male reproduction and possibly brain function
Source: BMC Evol Biol. 2011 Oct 12;11:298. doi: 10.1186/1471-2148-11-298 (PMC3214919; doi:10.1186/1471-2148-11-298)
Supplement: Additional file 8 — Table S3 The pairwise Ka/Ks ratios of RHOXF2 in the primate species tested. The Ka/Ks ratios were estimated following Pamilo-Bianchi-Li's method. [file 1471-2148-11-298-S8.DOC]

**Additional file 8.**

**Table S3 The pairwise Ka/Ks ratios of *RHOXF2* in the primate species tested.** The Ka/Ks ratios were estimated following Pamilo–Bianchi–Li's method.

|  | HUM-1 | HUM-2 | CHP-1 | CHP-2 | GOR | ORA | SIA | WBG | WCG | BLM-1 | GLM-1 | GLM-2 | BLM-2 | DL | YGM | RG | RM-1 | RM-2 | STM | PTM-1 | PTM-2 | AM |
| --- | --- | --- | --- | --- | --- | --- | --- | --- | --- | --- | --- | --- | --- | --- | --- | --- | --- | --- | --- | --- | --- | --- |
| HUM-2 | ∞ |  |  |  |  |  |  |  |  |  |  |  |  |  |  |  |  |  |  |  |  |  |
| CHP-1 | 4.1723 | 5.0826 |  |  |  |  |  |  |  |  |  |  |  |  |  |  |  |  |  |  |  |  |
| CHP-2 | 2.8698 | 3.1638 | 1.0249 |  |  |  |  |  |  |  |  |  |  |  |  |  |  |  |  |  |  |  |
| GOR | 1.4382 | 1.5858 | 2.8484 | 2.0624 |  |  |  |  |  |  |  |  |  |  |  |  |  |  |  |  |  |  |
| ORA | 1.5149 | 1.4946 | 2.1550 | 1.8165 | 0.9612 |  |  |  |  |  |  |  |  |  |  |  |  |  |  |  |  |  |
| SIA | 1.8728 | 2.0431 | 2.8142 | 2.5135 | 1.4719 | 1.3098 |  |  |  |  |  |  |  |  |  |  |  |  |  |  |  |  |
| WBG | 1.8112 | 1.9668 | 2.6267 | 2.3750 | 1.4439 | 1.2250 | 1.2122 |  |  |  |  |  |  |  |  |  |  |  |  |  |  |  |
| WCG | 1.7179 | 1.7824 | 2.6773 | 2.4195 | 1.3839 | 1.2042 | 1.1622 | 0.3043 |  |  |  |  |  |  |  |  |  |  |  |  |  |  |
| BLM-1 | 1.2263 | 1.2589 | 1.4254 | 1.5028 | 1.1885 | 1.0354 | 1.2193 | 1.2696 | 1.2355 |  |  |  |  |  |  |  |  |  |  |  |  |  |
| GLM-1 | 1.3224 | 1.3550 | 1.5023 | 1.5838 | 1.1814 | 1.1263 | 1.3216 | 1.3732 | 1.3337 | 1.0059 |  |  |  |  |  |  |  |  |  |  |  |  |
| GLM-2 | 1.2315 | 1.2168 | 1.3826 | 1.4489 | 1.1011 | 1.0268 | 1.3144 | 1.3390 | 1.3012 | 2.2729 | 3.4993 |  |  |  |  |  |  |  |  |  |  |  |
| BLM-2 | 1.1319 | 1.1180 | 1.2992 | 1.3619 | 1.0866 | 0.9207 | 1.1944 | 1.2192 | 1.1873 | 3.2950 | 4.3604 | 1.2326 |  |  |  |  |  |  |  |  |  |  |
| DL | 1.2902 | 1.3257 | 1.4200 | 1.5005 | 1.1489 | 1.0949 | 1.2992 | 1.3511 | 1.3072 | 1.3786 | 1.6249 | 1.9030 | 1.6588 |  |  |  |  |  |  |  |  |  |
| YGM | 1.5132 | 1.5512 | 1.6803 | 1.7863 | 1.3631 | 1.2498 | 1.5121 | 1.5681 | 1.5122 | 2.3667 | 2.6962 | 2.3969 | 2.0943 | 6.5441 |  |  |  |  |  |  |  |  |
| RG | 1.2132 | 1.2416 | 1.3399 | 1.4053 | 1.0935 | 1.0904 | 1.1787 | 1.1756 | 1.1480 | 1.5381 | 1.8016 | 1.7494 | 1.4867 | 1.7533 | 2.8539 |  |  |  |  |  |  |  |
| RM-1 | 1.0797 | 1.1107 | 1.1998 | 1.2577 | 0.9204 | 0.9541 | 1.1975 | 1.1665 | 1.1354 | 1.4711 | 1.7762 | 1.7911 | 1.4176 | 1.7011 | 3.1237 | 0.9652 |  |  |  |  |  |  |
| RM-2 | 1.0222 | 1.0516 | 1.1304 | 1.1818 | 0.8705 | 0.9549 | 1.2547 | 1.2231 | 1.1896 | 1.9119 | 2.2237 | 1.7938 | 1.4192 | 2.3216 | 3.9320 | 1.2973 | ∞ |  |  |  |  |  |
| STM | 0.9923 | 1.0198 | 1.0947 | 1.1377 | 0.8446 | 0.8719 | 1.0720 | 1.1045 | 1.0785 | 1.1003 | 1.3101 | 1.3206 | 1.0679 | 1.1455 | 2.0423 | 0.7833 | 0.2898 | 0.6218 |  |  |  |  |
| PTM-1 | 1.0093 | 1.0379 | 1.1113 | 1.1535 | 0.8574 | 0.9385 | 1.2219 | 1.1922 | 1.1613 | 1.7381 | 2.0104 | 1.6368 | 1.3119 | 2.0144 | 3.2398 | 1.2211 | 3.5685 | 0.5417 | 0.6050 |  |  |  |
| PTM-2 | 1.0874 | 1.1189 | 1.2077 | 1.2604 | 0.9221 | 0.9557 | 1.1999 | 1.2221 | 1.1887 | 1.4716 | 1.7766 | 1.7911 | 1.4180 | 1.7017 | 3.1245 | 0.9664 | ∞ | ∞ | 0.6243 | 3.5659 |  |  |
| AM | 1.0365 | 1.0657 | 1.1458 | 1.1927 | 0.8816 | 0.9916 | 1.2412 | 1.2094 | 1.1765 | 1.6085 | 1.9146 | 1.9310 | 1.5544 | 1.8971 | 3.3800 | 1.0677 | ∞ | ∞ | 0.0000 | 2.5958 | ∞ |  |
| PDM | 1.0365 | 1.0657 | 1.1458 | 1.1927 | 0.8816 | 0.9916 | 1.2412 | 1.2094 | 1.1765 | 1.6085 | 1.9146 | 1.9310 | 1.5544 | 1.8971 | 3.3800 | 1.0677 | ∞ | ∞ | 0.0000 | 2.5958 | ∞ | 0.0000 |
