# Supplementary material for: Rapid evolution and copy number variation of primate RHOXF2, an X-linked homeobox gene involved in male reproduction and possibly brain function
Source: BMC Evol Biol. 2011 Oct 12;11:298. doi: 10.1186/1471-2148-11-298 (PMC3214919; doi:10.1186/1471-2148-11-298)

**Additional file 9.**

**Figure S6 The expression pattern of *RHOXF2* determined by qPCR in two extra rhesus macaque individuals.** (a) a 2yr male; (b) a 2yr female. The relative expression levels were calculated by setting the value in the testis as “1”. The result is consistent with the data presented in Figure 5.


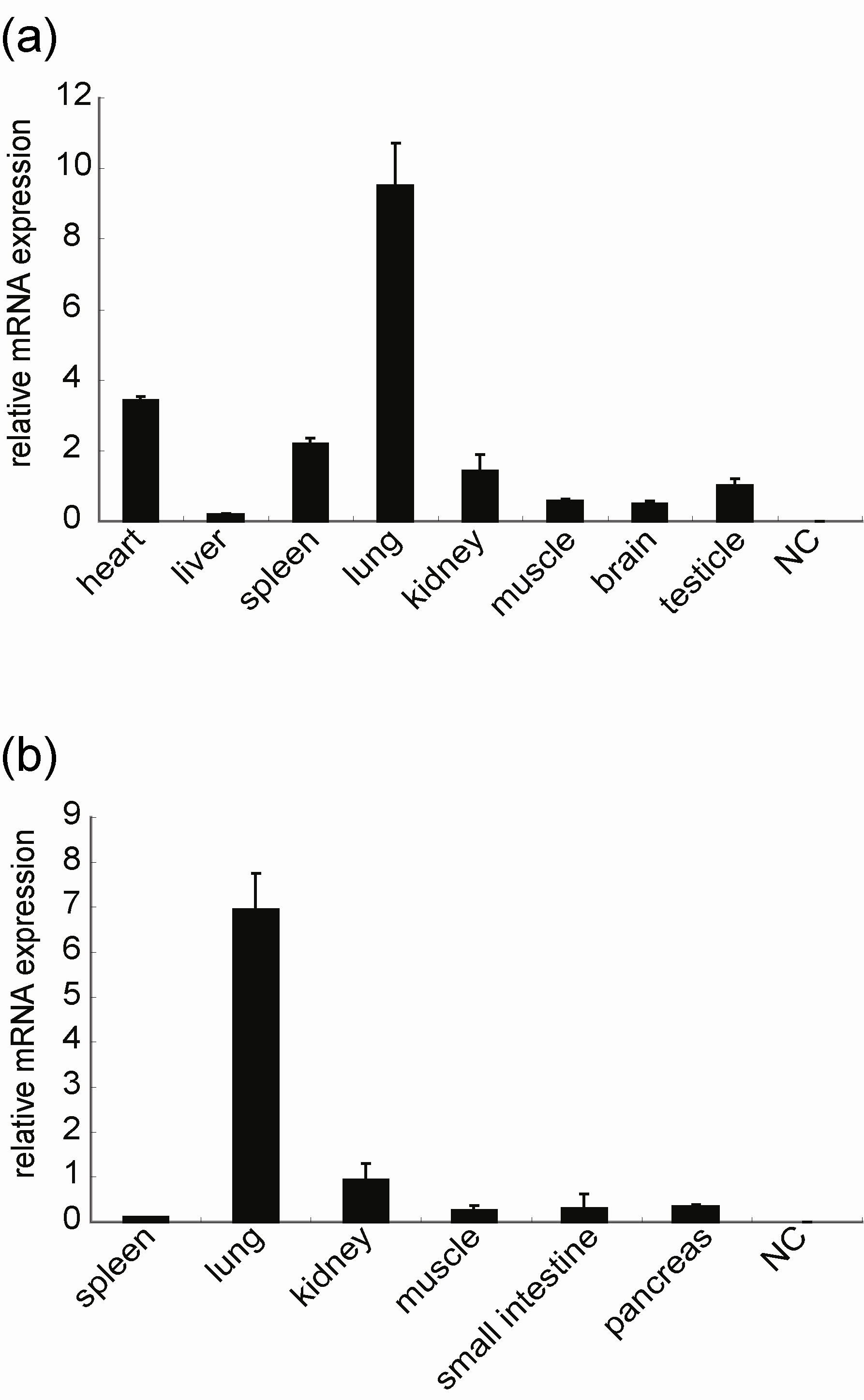

Supplement: Additional file 9 — Figure S6 The expression pattern of RHOXF2 determined by qPCR in two extra rhesus macaque individuals. (a) a 2 yr male; (b) a 2 yr female. The relative expression levels were calculated by setting the value in the testicle as "1". The result is consistent with the data presented in Figure 5. [file 1471-2148-11-298-S9.DOC]
