# Supplementary material for: Rapid evolution and copy number variation of primate RHOXF2, an X-linked homeobox gene involved in male reproduction and possibly brain function
Source: BMC Evol Biol. 2011 Oct 12;11:298. doi: 10.1186/1471-2148-11-298 (PMC3214919; doi:10.1186/1471-2148-11-298)
Supplement: Additional file 10 — Table S4 The primer sequences of PCR and sequencing in this study. [file 1471-2148-11-298-S10.DOC]

**Additional file 10.**

**Table S4 The primer sequences of PCR and sequencing in this study.**

|  | Primer name | Sequences (5’-3’) |
| --- | --- | --- |
| *RHOXF2* genomic DNA primer | NM032498_E1-2F | TAGTGGACTTTCTCAAGGAGTGTGC |
|  | NM032498_E1-2R | TTCTGCCTCGCCTGGGATAG |
|  | NM032498_E1-2F2 | AGCAAATAGCGGGAAGCAGA |
|  | NM032498_E1-2R2 | GAGGGACTGCGGGAAAGC |
|  | NM032498_E1-2F3 | AGCAAATAGCGGGAAGCAGA |
|  | NM032498_E1-2R3 | ACCCAGACGAACGACGGACT |
|  | NM032498_E3F | AATTAAACACAAGAATGAAACGACA |
|  | NM032498_E3R | ATGGAAGTGCTACCTAAAGGATACC |
|  | NM032498_E3R2 | CAAAGTTCAGACCTCATTGCCTATA |
|  | NM032498_E4F | GAAAAGGTTAAGGACATAGAGAGTTGC |
|  | NM032498_E4R | AGAATGTCTGGGTCGGTGTAAGC |
|  | MAR-e4-1-F | CTTTATGCTGAGGATGGGCTTTT |
|  | MAR-e4-1-R | TGATGCTGACTCAAGGAGATTACTG |
|  | MAR-e4-2-F | GGGACCAAGTCATCGGCTACA |
|  | MAR-e4-2-R | TGATGGGGTGCTATGCAAAAT |
|  | MAR-e4-seq-F | CAGGGGTGGTATGGAAGATGG |
|  | MAR-e4-seq-R | AGAAATGGACAATAACAAAAAAGAATG |
|  | MAR-e1e2-1-F | CCCGCCGAGGAGATAGTAGGA |
|  | MAR-e1e2-1-R | TGGTTCAGGAGGGGATTTAGG |
|  | MAR-e2-1-F | AGAGGCTAGTGGACTGGACGG |
|  | MAR-e1-1-R | GCTGTGCTCATCGTTGCCATT |
|  | MAR-e1-2-F | GCAGTTCCAACCCACAGCAA |
|  | MAR-e1-2-R | CTACAGGCGTACCACCGTCC |
|  | MAR-e3-1-F | CTGGGTGTAGGGGAGTTAGTGG |
|  | MAR-e3-1-R | TTCCCGTGGATAGTCATGTTCC |
|  | MAR-e3-2-F | GTAAACCATTTCTAAGGCAACCATG |
|  | MAR-e3-2-R | GGAACGGGAAAGAGGAAAGGA |
|  | MAR-e3-4-F | TGGCTCATCACTTTCATATCGG |
|  | MAR-e3-4-R | AGTAGTTCAGTTCTATTTTCTTTGGTCTTT |
| *RHOXF2* cDNA primer | nm032498_cDNA_reverseT | TTGAGAACTTACTGACCCCATGTG |
|  | NM032498_cDNA_F | GGCGGCGACTCACAGTTCTA |
|  | NM032498_cDNA_R | TTACTGACCCCATGTGACACACAA |
|  | pepp2-rm-cDNAF | GCGGTGCTGTCGCCTACTGA |
|  | pepp2-rm-cDNAR | GTGAAAGAATGCCCGATAACAAAAG |
|  | NM032498_cDNA_F2 | CAGCAAATAGCGGGAAGCAG |
|  | NM032498_cDNA_R2 | TGTGAAAGAAGGCACGATAACAA |
|  | nm032498_cDNA_F6 | AGGCACAGCAGCAGGAGAAA |
|  | nm032498_cDNA_R6 | ATGAGTGGAAGGGGCAAGGA |
|  | pepp2-cDNA-7-F3 | CGGACCAGTGTAGCCAGTA |
|  | pepp2-cDNA-7-R3 | ATGTGAAAGAAGGCACGATA |
| Real-time PCR and RT PCR primer | NM032498_RT_F | AGGGCATCAATGGCAAGAAAC |
|  | NM032498_RT_R | AGGCTGCTGGAATGGCTGT |
|  | NM012253_RT_F | TGGCAATCTTTGATGTGAACCG |
|  | NM012253_RT_R | GGGGCAGGACAGAATGGAAAT |
|  | PEPP2-RT-owm-F | AGAAGAGCCAAGTGGAGGAGACA |
|  | PEPP2-RT-owm-R | GCAGTTACCATGACAGGCTGG |
|  | TKTL1-RT-owm-F | CTACCGGGTGTTCTGCCTCAT |
|  | TKTL1-RT-owm-R | AGATTGTCCAGACTGTAGTAGGAAGCA |
|  | pepp2-GB-QF3 | ACTACAGGATATGAATGCTGCGGT |
|  | pepp2-GB-QR3 | TGCTGCTTCTGTGCCTTGCT |
|  | pepp2-HUM-QF1 | CGTCCACGCCTTCACCCC |
|  | pepp2-HUM-QR1 | GTCTCCTCCATTTGGCTCTTCTATT |
|  | pepp2-RM-QF2 | CAGGAGCTGGAGCGCATTTTC |
|  | pepp2-RM-QR2 | CCTCCACTTGGCTCTTCTATTCTCA |
|  | PEPP2-cRT-chp-hum-F1 | CGAGCAGTTCCCCAGTGAGTT |
|  | PEPP2-cRT-chp-R1 | CCATTGATGCCCTCTGATGTCTC |
|  | PEPP2-RT F01 | AACTCGCAGTGCAGATTTGGT |
|  | PEPP2-RT R01 | TGCTGGAATGGCTGTGGTC |
|  | GAPDH_F | ATTGCCCTCAACGACCACTTT |
|  | GAPDH_R | GGTCTCTCTCTTCCTCTTGTGCTCT |
| Breakpoint & unsure sequence primer | NM032498_P1F | ACATAAGCCAGGAAGAAGAGG |
|  | NM032498_P1R | CCCTTGGGACAGTTGGAGTTC |
|  | NM032498_P2F | CTCAATAATGGAACCACGAAAG |
|  | NM032498_P2R | TTTAATTGACTCAGGTACGCAGG |
|  | NM032498_D1F | GAGGGAAAGTGCACTGCAAGGG |
|  | NM032498_D2R | TTTTGGACTAAGTTATTACAACAGAG |
|  | 349N3_bp1_F1 | ATGATGACCAGGGGCAGATACA |
|  | 349N3_bp1_R1 | TTGCTCCATTAAGGTGACTACGG |
|  | 349N3_bp1_F2 | AATGGGCCATTGGAAAGATGA |
|  | 349N3_bp1_R2 | TTGTCACTAGGTGGCGCAAAA |
|  | 349N3_bp1_F3 | AGGGAATATGACACTGGAGTTTGG |
|  | 349N3_bp1_R3 | TTGTGCATGGGTAACAGAGGAGT |
|  | 349N3_bp2_F1 | CGAGGGAGTGGGAACAGTAGATG |
|  | 349N3_bp2_R1 | CCAGGTGAAAGATAATGGGCAGT |
|  | 349N3_bp2_F2 | GGGCTCCTACTAACCTCCGTTGT |
|  | 349N3_bp2_R2 | TCACCTAAAAGCCGAATCTACAAAT |
|  | 349N3_bp2_F3 | GCTCAATGCGTAATACCACCTTT |
|  | 349N3_bp2_R3 | ACGGAGTCTCACTCTGTCGTCC |
|  | 349N3_bp3_F1 | GTGATGTCACCAAAGATGGAGTAGA |
|  | 349N3_bp3_R1 | GGAAGTCCCAGAAGGTAAGGCT |
|  | 349N3_bp3_F2 | CAACGGGCAAATCGCACAG |
|  | 349N3_bp3_R2 | CCATTCCTTGACATTCATCCACATA |
|  | 349N3_bp3_F3 | AGACAAGATCCTAAAAGCAGCAAGA |
|  | 349N3_bp3_R3 | TGTGAACCAAGTGGCATCAGC |
|  | 349N3_bp4_F1 | TGCCCTCTTCTGGACTATCTGG |
|  | 349N3_bp4_R1 | CGCCTCTGCATTGGGTAAATT |
|  | 349N3_bp4_F2 | TGACTGTGATCCCCACAAACC |
|  | 349N3_bp4_R2 | CGATCTTGGCTCACTGCAACC |
|  | 349N3_bp4_F3 | TGCCATCATTGCTGAGCTTAAG |
|  | 349N3_bp4_R3 | AAGTTATTACAACAGAGGCTACTCTTCG |
|  | 349N3_dp2_F001 | CAGCAAATAGCGGGAAGCA |
|  | 349N3_dp2_R001 | ACTGTGGGAAAGCCAAACG |
|  | 349N3_dp2_F002 | CCACCTCCTTCCAACACCC |
|  | 349N3_dp2_R002 | TGCCCAGGTTCCTTCTCG |
|  | 281C1_ins_F001 | GTAGACATGGGAGACTTTTCATTTTG |
|  | 281C1_ins_R001 | CATTCTTGGGTGTTTCTCACAGAG |
|  | 281C1_ins_F002 | ATCGGATGGTTGCCGTGTCT |
|  | 281C1_ins_R002 | TGGGTACTTGAGATTAGGGAGTGGT |
|  | 281-565-1F | GGGCATTGACCCTATCTAGTGAGA |
|  | 281-565-1R | AACCATCGGTCCTCCTGTTTATC |
|  | 281-565-2F | CTGTTCAAGACAGCCAAGCAAA |
|  | 281-565-2R | AAATCCATAGACAGAAGTTAAAGGACTC |
|  | 349-565-1F | GTCTCAAAACTGAAGAACTTGGGAGT |
|  | 349-565-1R | CCACAAGTCTGACATACCAGGATAAA |
|  | Front100k-F | CTCTTTGCTTTGAGTCCCAATAACC |
|  | Front100k-R | GCCACCTATGCCTCGTCTGC |
|  | Front50k-F | CCATCAGGCAGGCACTGTTAT |
|  | Front50k-R | TGCTTCTAGGGCTTTCTTAGGC |
|  | Front150k-F | CTTGTGGAGGGGAAGTATTGGG |
|  | Front150k-R | AAACCTTTGAAGTAAGCAGCAGTG |
|  | Back100k-F | ACAGATGAGCGAGGAGGCAAAG |
|  | Back100k-R | AAATGACCTAAGTTACAGCCAGAAAA |
|  | Back50k-F | CTCATCCACCCACTCCTCCG |
|  | Back50k-R | GCAATAAACTTGGGGTGCCATT |
|  | Back150k-F | CCTACTTTGGTTTGGTCGGTCT |
|  | Back150k-R | TGAGCAGTGGGCATGGATTTA |
| BAC sequence variation  primer | 565-281 v1 F01 | GGTCCTCCTGTTTATCCC |
|  | 565-281 v1 R01 | GTTCCCACTCCCTCGTAG |
|  | 565-281 v1 F02 | TGGAGACCCTGTAAACTGACTTGTA |
|  | 565-281 v1 R02 | CAGCATGAACAAACCCGTAAATC |
|  | 565-281v11F01 | AAGCAACTCTAAAGCCAGCAA |
|  | 565-281v11R01 | TGTTCAAGACAGCCAAGCAA |
|  | 565-281v11F02 | TCAAAACCGTGGAGCAATAG |
|  | 565-281v11R02 | TGCTAAGCAGAGTAGCTGATGTC |
|  | 565-349 V2 F01 | TGGGAGGATTTGTTGTTAG |
|  | 565-349 V2 R01 | CACTTTGGGTCTGGTATTC |
|  | 565-349 V2 F02 | CAGCCACTCTGGGTATGACAGTT |
|  | 565-349 V2 R02 | TTCCCTAGAGGGACAGAACAAATAG |
